# Modeling of the Dorsal Gradient across Species Reveals Interaction between Embryo Morphology and Toll Signaling Pathway during Evolution

**DOI:** 10.1371/journal.pcbi.1003807

**Published:** 2014-08-28

**Authors:** Priscilla Ambrosi, Juan Sebastian Chahda, Hannah R. Koslen, Hillel J. Chiel, Claudia Mieko Mizutani

**Affiliations:** 1 Department of Biology, Case Western Reserve University, Cleveland, Ohio, United States of America; 2 Department of Genetics and Genome Sciences, Case Western Reserve University, Cleveland, Ohio, United States of America; North Carolina State University, United States of America

## Abstract

Morphogenetic gradients are essential to allocate cell fates in embryos of varying sizes within and across closely related species. We previously showed that the maternal NF-κB/Dorsal (Dl) gradient has acquired different shapes in *Drosophila* species, which result in unequally scaled germ layers along the dorso-ventral axis and the repositioning of the neuroectodermal borders. Here we combined experimentation and mathematical modeling to investigate which factors might have contributed to the fast evolutionary changes of this gradient. To this end, we modified a previously developed model that employs differential equations of the main biochemical interactions of the Toll (Tl) signaling pathway, which regulates Dl nuclear transport. The original model simulations fit well the *D. melanogaster* wild type, but not mutant conditions. To broaden the applicability of this model and probe evolutionary changes in gradient distributions, we adjusted a set of 19 independent parameters to reproduce three quantified experimental conditions (i.e. Dl levels lowered, nuclear size and density increased or decreased). We next searched for the most relevant parameters that reproduce the species-specific Dl gradients. We show that adjusting parameters relative to morphological traits (i.e. embryo diameter, nuclear size and density) alone is not sufficient to reproduce the species Dl gradients. Since components of the Tl pathway simulated by the model are fast-evolving, we next asked which parameters related to Tl would most effectively reproduce these gradients and identified a particular subset. A sensitivity analysis reveals the existence of nonlinear interactions between the two fast-evolving traits tested above, namely the embryonic morphological changes and Tl pathway components. Our modeling further suggests that distinct Dl gradient shapes observed in closely related *melanogaster* sub-group lineages may be caused by similar sequence modifications in Tl pathway components, which are in agreement with their phylogenetic relationships.

## Author Summary

Embryo size can vary greatly among closely related species. How tissue specification either scales or is modified in the developing embryo in different species is an ongoing investigation in developmental biology. Here we asked how embryo morphology and specific molecular pathways influence tissue specification by altering the distribution of morphogens. Morphogens are molecules that form gradients that regulate gene expression patterns in a dosage-dependent fashion that result in tissue specification, and therefore are a prime target for evolution in order to adjust or maintain tissue proportions in relation to overall embryo size. We used a mathematical model to identify factors that influence the distribution of the Dorsal morphogen gradient that is responsible for patterning the dorsal-ventral axis of the *Drosophila* fruit fly embryo. We obtained experimental data from mutant conditions and different species of *Drosophila* to calibrate our model and found an interaction between embryo morphology and regulation of the Toll pathway, which regulates the Dorsal gradient. Furthermore, the model predicts that closely related species share similar modifications in Toll pathway components resulting in their species-specific gradient shapes, which are supported by interspecies amino acid comparison of the components Dorsal and Cactus.

## Introduction

The embryonic patterning and development of limbs rely on morphogenetic gradients that set up territories of gene expression in a dosage-dependent fashion [1], [2]. Rather than a static process, cell fate specification normally occurs under dynamically changing environments that involve cell divisions and tissue growth expansion. One important property of morphogenetic gradients is the ability to scale and accommodate tissue cell types despite fluctuations in organismal size, for instance, due to feeding conditions or mutations affecting growth. Scaling is also a fascinating problem in evolutionary biology and can be observed in related species that have dramatically changed in embryo size but kept fixed gene expression domains at relatively similar positions in relation to the whole body [3]. Recent quantitative studies have begun to elucidate the scaling mechanisms of morphogenetic gradients during tissue growth [4], regeneration [5], as well as in related species [6]–[8] or artificially selected strains of same species that vary in embryo size [9]–[12]. In particular, studies in *Drosophila* embryonic gradients stand out as being especially amenable to quantitative analysis and modeling [13]. The relatively simple syncytial organization of the embryo allows precise detection of target gene expression with single cell resolution, and models can be built based on the extensive biochemical data of signaling pathways responsible for gradient formation. Remarkably, the isolation of new closely related species to the *Drosophila melanogaster* model (reviewed in [14]) provides a rich natural repertoire of genetic variations in egg size, cell numbers and gene divergence, which can be used to test the impact of these evolutionary changes on the scaling of gradients.

Here we address the question of gradient scaling across related *Drosophila* species using the embryonic dorso-ventral (DV) patterning as a model system. The maternal nuclear concentration gradient of the NF-κB related transcription factor Dorsal (Dl) subdivides the embryo into three germ layers: the mesoderm, neuroectoderm and ectoderm. High levels of nuclear Dl in the ventral embryonic side activate expression of mesodermal genes, such as *snail* (*sna*), whereas moderate levels in lateral regions activate neuroectodermal genes. Low to negligible levels of nuclear Dl in dorsal regions allow the expression of ectodermal genes such as *decapentaplegic* (*dpp*) and *zerknult* (*zen*), due to the lack of repression that Dl exert on these genes (reviewed in [14]).

**Figure 1:**
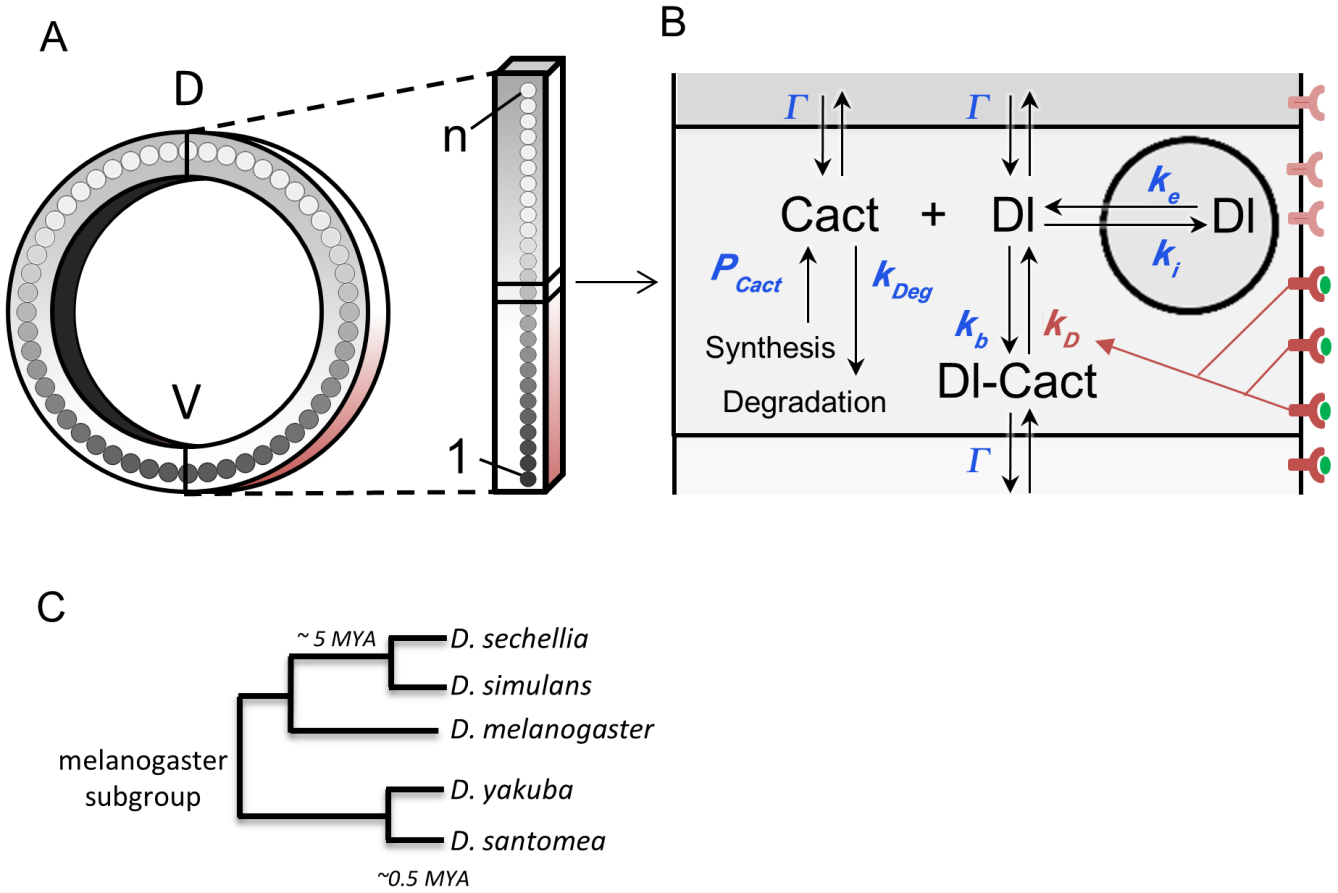
Dl gradient model rationale. A) The embryo is modeled as a single string of *n* cuboid cellular compartments. B) Chemical reactions and transport processes considered by the Kanodia model [15]. The DV Toll receptor activation gradient (red) is represented by the space-dependent Dl-Cactus dissociation constant (*k_D_*) and results in higher nuclear concentrations of Dl (gray) at the ventral side of the embryo (V) and higher cytoplasmic concentrations at the dorsal side (D). Parameters shown in blue are explained in Table S2. C) Phylogenetic tree of *melanogaster* subgroup species.

We recently reported that the Dl gradient has unique distribution profiles in related Drosophilids that vary in embryo size, which result in unequally scaled germ layers [8]. For instance, changes in mesodermal size serve as a mechanism to specify the border of the neuroectoderm and keep it at a constant size. Here we combined experimental approaches and mathematical modeling in order to identify parameters that might be responsible for the modified distributions of Dl gradient across species. Previously, Kanodia *et al*. (2009) [15] developed a mathematical model for *D. melanogaster* that reproduces the dynamics of the Dl gradient formation during cleavage cycles (Fig. 1A, B). Their model consists of differential equations derived from mass balance equations of the main biochemical interactions of the Toll (Tl) pathway that lead to Dl transport into the nucleus, which were numerically solved using globally optimized parameters. Briefly, the model simulates the graded nuclear translocation of Dl initiated by the space-dependent dissociation of the cytoplasmic complex formed between Dl and Cactus/Ik-B (Cact). This dissociation is modeled by a reaction rate constant *k_D_* and represents the graded activation of Tl receptors along the embryonic DV axis. The Dl-Cact complex prevents Dl from entering the nucleus and its dissociation due to Tl activation frees Dl to enter the nucleus. The model also recreates the geometric arrangement of embryonic nuclei during cleavage cycles, as well as changes in nuclear surface area, which affect Dl nuclear import and export rates. The Kanodia model captures essential properties of the Dl gradient formation and correctly reproduces the dynamics of the gradient formation during early embryonic cycles. However, this model has not yet been formally validated in conditions other than wild type *D. melanogaster* embryos, or used to simulate the Dl gradient of other species.

Kanodia *et al.* (2009) [15] employed a genetic algorithm to identify a cloud of dimensionless parameters that satisfied a small dataset of experimental Dl gradient measurements from wild type embryos only. In this work, we built upon this model, and attempted to validate its generality by fixing free parameters using biological measurements, and manipulating only the subset of parameters that were most likely to be biologically relevant. We manipulated a single representative parameter set from this model in order to identify which parameter changes are sufficient to reproduce the experimental Dl gradients from three distinct experimental conditions in *D. melanogaster*: (1) embryos with decreased Dl levels, (2) decreased nuclear size with high nuclear density, and (3) increased nuclear size with low nuclear density. Once we obtained adjusted parameters for *D. melanogaster* that also satisfied these extended conditions, we next asked which parameters from this representative set were most likely to be modified in *Drosophila* species that display distinct Dl gradient shapes. To this end, we selected a divergent species with small embryos, *Drosophila busckii*, and two additional pairs of species belonging to the *melanogaster* subgroup, *Drosophila simulans/Drosophila sechellia* and *Drosophila santomea/Drosophila yakuba*, which diverged from *D. melanogaster* between 5 to 6 MYA (Fig. 1C) [14], [16], [17]. These species give us the unique opportunity to assay the behavior of the Dl gradient in lineages that have undergone a separate speciation event, but share some commonalities. For example, *D. sechellia* and *D. santomea* diverged very recently from their ancestral siblings *D. simulans* and *D. yakuba*, respectively, at an estimated 0.3–0.5 MYA. Despite such short divergence time, *D. sechellia* and *D. santomea* have much larger embryos than their siblings [6]. The use of modeling gave us insights in the evolution of Dl gradient shapes that are in agreement with the phylogenetic relationships of the species analyzed. We show that although the modified embryonic anatomy of these species influence the Dl gradient distribution, the species-specific Dl gradient shapes also depend on genetic modifications in the Tl pathway, which are shared in closely related species pairs.

## Results

### Reconstruction of the Kanodia model reproduces the Dl gradient shape in some mutant conditions

We are interested in understanding how the Dl gradient acquired distinct shapes in related *Drosophila* species. One notable phenotypic difference reported in several *Drosophila* species is the significant variability in egg size [6], [18]. In addition, the nuclear size and density also vary in these species [19]. We previously showed that manipulations in nuclei size and density in mutant *D. melanogaster* embryos can recreate Dl gradient shapes that are found in nature, leading us to hypothesize that nuclei density and size changes might be sufficient to modify the Dl gradient shape.

**Figure 2:**
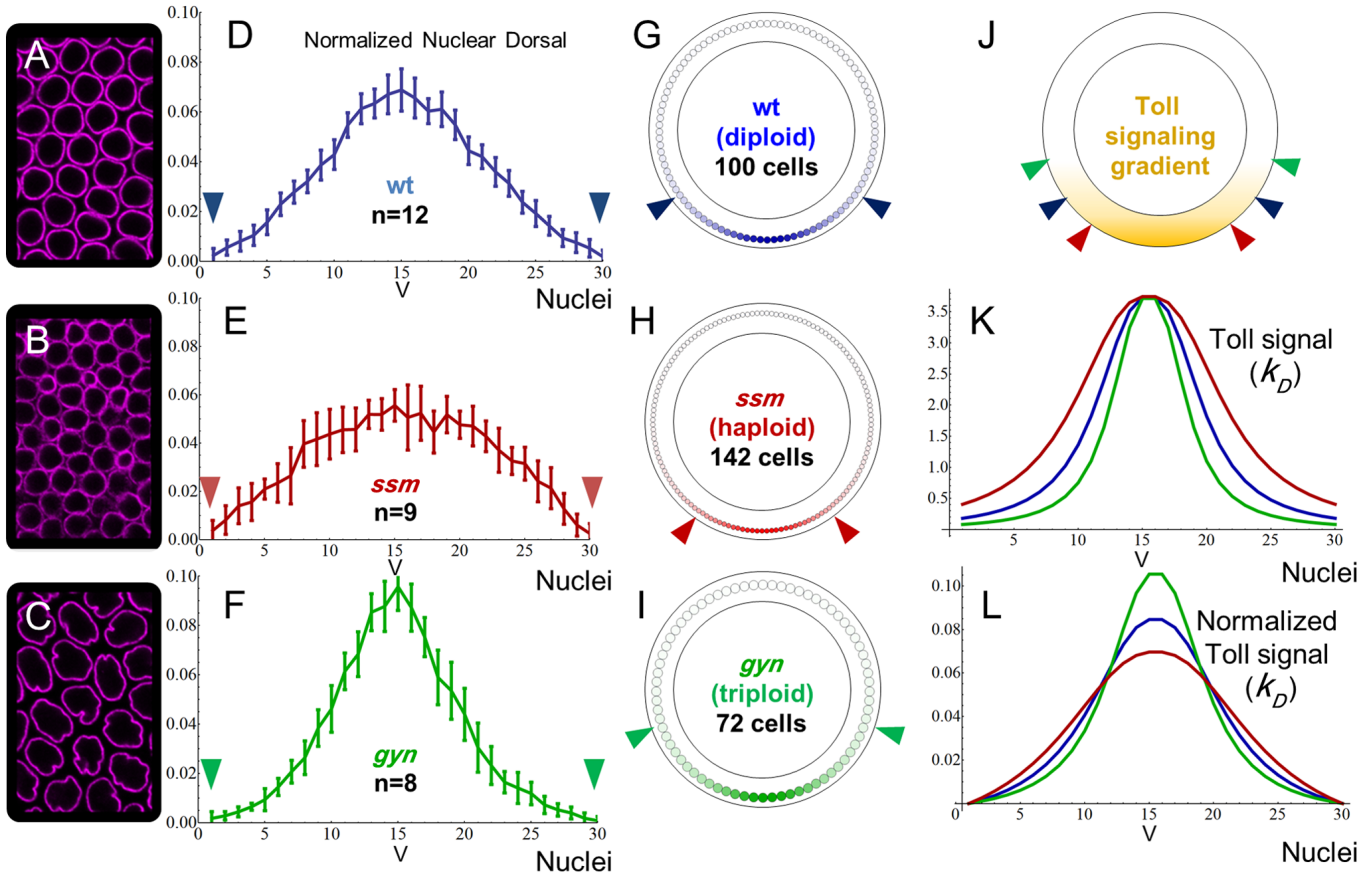
The Dl gradient is modulated by changes in nuclear size and density. A–C) Increasing nuclear size and decreasing nuclei density from haploids (*ssm,* B) to diploids (A) to triploids (*gyn,* C) stained with anti-laminin (magenta). D–F) Normalized graphs showing distinct Dl gradient shapes from *D. melanogaster* (D), *ssm* (E) and *gyn* (F, mean±SD). G–J) Cross-section schemes for wild type (G), *ssm* (H) and *gyn* (I), and a *D. melanogaster* embryo (J) representing the Toll signaling gradient. K) Simulated Toll signaling gradient based on the equation for *k_D_*, the space-dependent Dl-Cactus dissociation constant. As illustrated in (J), nuclei density affects the angle subtended by 30 cells in a cross-section, resulting in a larger rate of Toll signal decay for *gyn* and a smaller rate for *ssm*. (L) Normalized Toll signaling gradients, emphasizing the relationship between the simulated Toll signaling gradient and experimental Dl gradients. Figures (A–F) were modified from [8]; **V**, ventral midline; color-coded arrowheads in D–J delimit the 30 ventral most cells.

The mutation *sesame* (*ssm*) generates haploid embryos that undergo an additional round of mitotic division, causing a high nuclear density and decreased nucleus size (Fig. 2A, B) [20]. In these mutants, the Dl gradient becomes flattened (Fig. 2D, E). The second mutation used, *gynogenetic-2, gynogenetic-3* (referred to as *gyn* [21]), generates triploid embryos that stop dividing one cycle earlier causing a lower nuclear density and larger nucleus size compared to the wild type diploid embryos (Fig. 2C). The Dl gradient of *gyn* embryos is sharper than the wild type (Fig. 2F). These mutations are known to affect ploidy, but are not expected to alter components in the Tl signaling pathway or embryo size. One way of explaining the altered Dl gradients of these mutants would be if the density of nuclei modifies the reading of Tl signal from one nucleus to the next, and consequently the rate of Tl signal decays (Fig. 2G–J). In this scenario, a lower versus higher nuclei density would lead to a steeper and a flatter gradient, respectively (Fig. 2L). In addition, since these mutants have the same amount of maternal Dl protein, the increase in nuclei density would decrease the amount of Dl per cell compartment and flatten the gradient. Another consideration is the differences in nuclei size, which increases the surface area available for Dl transport. Thus, nuclear size may counterbalance the effects of nuclei density.

Since a qualitative analysis would not be sufficient to predict all of the combined effects described above, we employed a numerical approach using a modified version of the Kanodia model (Text S1). We used the same values of a representative parameter set used in the original MATLAB code to reconstruct the original model and run simulations of the wild type gradient formation using Wolfram's Mathematica, which successfully reproduced key features of the model (Details of state variables, equations and parameters are provided in Text S1 and Tables S1, S2, S3). In principle, any set within the restricted cloud of parameter sets identified by Kanodia et al. [15] could be used to model the Dl gradient and investigate qualitative changes to simulate the mutant gradients. We then asked if the shape of *ssm* and *gyn* gradients were altered from the onset of the Dl gradient formation, at nuclear cycle 10 (nc10). One of the Kanodia model findings was that the wild type Dl gradient has a constant shape throughout the nuclear cycles, which matches experimental data [15]. We initially tested the effect of changing nuclear radius in the wild type from nc10 to nc13 over the final gradient shape at the last stage (nc14). We found that altering the size of nuclei modifies the shape of the Dl gradient at early stages, but does not affect its final shape at nc14 (Fig. S2). Since we are most interested in the gradient shape at the final cycle nc14, and not the dynamics of the mutant gradients, this result indicates that the effect of incorrect assumptions about early cycles is minimized.

### Simulations of nuclei numbers and size can reproduce *ssm* gradient, but not *gyn* gradient

**Figure 3:**
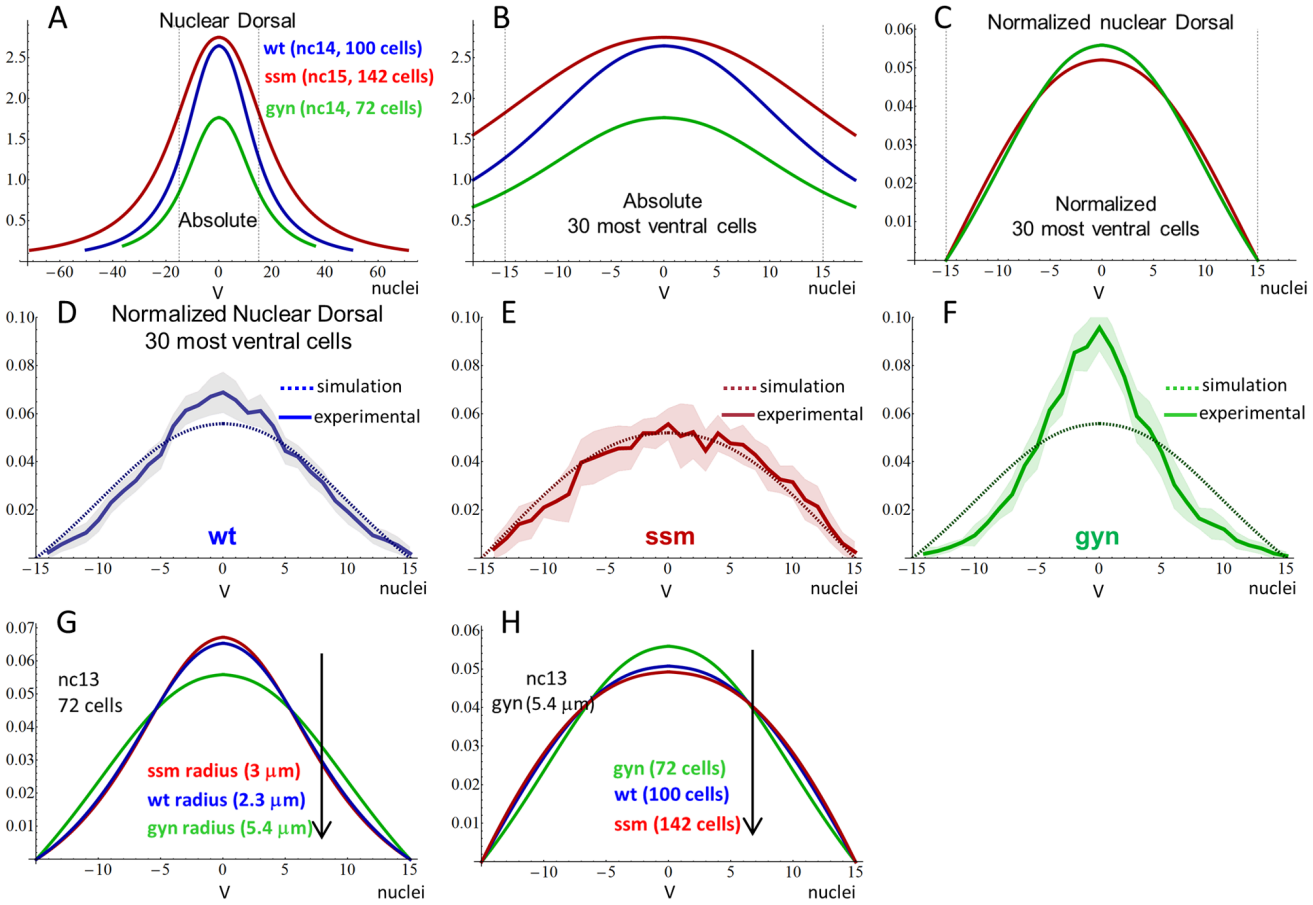
Comparison between experimental data and model output. A) Simulations of nuclear Dl levels at the last nuclear cycle of each genotype for the entire cross-section. Note that the cross-section has the same size in all genotypes, but the number of nuclei changes, due to extra or fewer nuclear cycles. B) Non-normalized and (C) normalized simulated gradients considering the 30 most ventral cells only. D–F) Direct comparison between experimental data and the model normalized output. Experimental data indicated by solid lines was reproduced from [8]. Shaded areas represent average±SD. G–H) Individual effects of changing nuclei radius and density on the Dl gradient at nuclear cycle 13. G) Increasing nuclei radius and (H) density flattens the gradient, as indicated by arrows. **V** indicates ventral midline, *y* axis indicate absolute (A, B) and normalized Dl levels (C–H), *x* axis indicate nuclei.

We next attempted to reproduce the Dl gradients from *ssm* and *gyn* mutants by using the selected representative set of parameters from Kanodia *et al.* [15] and adjusting it for nuclei size and density according to our experimental measurements (Fig. 3; Table S4). Few additional parameters were changed, especially related to early cycles (Text S2), but given the model robustness these changes did not significantly affect our results. We also normalized the model output to match our experimental data (see Methods), which is restricted to the 30 most ventral cells instead of the entire embryonic cross section (Fig. 3A–C). This ventral region includes the entire mesoderm and few additional cells in wild type and mutant embryos, and encompasses reliably measurable levels of nuclear Dl with distinguishable signal from background noise. This also represents the region where significant variations in the gradient shape are present [8]. With our normalization, we represent the overall shape of the Dl gradient instead of absolute values of Dl concentration (Fig. 3D–F). Unless otherwise noted, the normalized gradient restricted to the 30 most ventral cells is referred to as “Dl gradient”. Given the graded levels of nuclear Dl, we also verified that the variations in the net numbers of mesodermal cells between wild type and mutant embryos do not alter the overall shape of the gradients after normalization (Fig. S3).

In non-normalized graphs, our simulations show that *ssm* embryos have the highest peak of nuclear Dl concentration, while *gyn* embryos have the lowest peak (Fig. 3B). Thus, even though *ssm* has a smaller amount of Dl per cell compartment and nuclear surface area available for Dl translocation, the model predicts that its smaller nuclear volume is the major determinant of the absolute concentration of nuclear Dl. In terms of Dl gradient shape seen in normalized graphs, the model correctly reproduces the flattened *ssm* gradient, but not the steep *gyn* gradient, which instead appears with the same shape as wild type (Fig. 3E–F).

The fact that the model can reproduce the *ssm* but not the *gyn* gradient points to two non-exclusive deductions: (1) changes in nuclei density and size are sufficient to explain the *ssm* distorted gradient, but not *gyn*, i.e. our hypothesis is only partially correct; and (2) the parameter set used creates a strongly artificial robustness, buffering the effect of our manipulations. To investigate if our manipulations were being buffered, we first tested the individual effects of nuclei density and size on the Dl gradient shape. We found that either higher nuclei density or larger nuclear size result in a flattened gradient (Fig. 3G, H), indicating that the flattened *ssm* gradient is mostly determined by its higher nuclei density, which overrides the effect of its smaller nuclei. In contrast, the effect of larger nuclei in *gyn* was only slightly compensated by its reduced nuclei density, resulting in a Dl gradient shape similar to wild type in our simulations, rather than the steep gradient obtained experimentally.

The results above suggest that some of the assumptions that apply to wild type and *ssm* may not apply to *gyn*. One possibility is that one or more general parameters, such as Dl diffusion rates and Dl nuclear export rates are different from the values employed in the model, but they have a more significant effect under the *gyn* conditions. We also observe that the wild type simulation is not completely satisfactory, suggesting that this representative parameter set used could be further improved. We next modified the model to determine which parameter combinations could better reproduce our experimental Dl gradients.

### Refinement of the parameter values reveals that embryo geometry plus Dl diffusion and export rates play major roles in the model reproduction of the *gyn* gradient

To increase the model flexibility and allow testing the effects of individual parameter changes, we used dimensionalized equations and focused on the simulation at the last nuclear cycle only (see Methods). In the original model, 9 dimensionless parameters were used (Table S3, Text S1), in addition to nuclei radius and density, developmental timing and cell compartment volume at nc14. In our modified model, a total of 19 parameters can be manipulated independently (Table 1), and their effects on the Dl gradient shape can be directly analyzed (Fig. S5). The original values of most of these 19 parameters could be estimated from the representative non-dimensionalized parameter set chosen here, while others were determined by direct measurements and assumptions (Text S3). Revisions of the parameter values from this set were performed by manually testing a combination of parameters able to reproduce the gradients from wild type, *ssm,* and finally *gyn*. To further validate our revised parameter set, we also quantified Dl from *D. melanogaster* embryos derived from *dl^−^/dl^+^* heterozygote mothers (referred to as *dl^−^/dl^+^* embryos for simplicity) and tested the model ability to reproduce this mutant Dl gradient. These embryos have normal embryo size and Tl signaling, but only half of normal Dl protein amount.

**Table 1:** Parameter values used in model simulations for *D. melanogaster* wild type and mutant conditions shown in Figures 4 and 5.

	*mel* original parameters	*mel* adjusted parameters	*dl−/dl+* increased *k_i_*, *k_Deg_*	*dl−/dl+* increased *k_i_*, *k_Deg_*, *Г*	*ssm* 1 pink fig. 5	*ssm* 2 black fig. 5	*gyn* 1 increased *Г*	*gyn* 2 increased *Г* and *k*_e_	*gyn* 3 increased *Г, k_e_, Er*
Embryo 1/2 length (*El*; µm)	245	241.5	241.5	241.5	241.5	241.5	241.5	241.5	241.5
Embryo radius (*Er*; µm)	90	102.4	102.4	102.4	102.4	102.4	102.4	102.4	117
Cortical layer (*Eh*; µm)	31	25	25	25	30	30	29.9	29.9	29.9
Total nuclei (*Tn*)	6000	6000	6000	6000	12000	12000	3000	3000	3000
*R*	15340	15000	15000	15000	15000	15000	15000	15000	15000
*S*	4052	4000	4000	4000	4000	4000	4000	4000	4000
*ξ*	2.38	2.5	2.5	2.5	2.5	2.5	2.5	2.5	2.5
*Γ*	0.03	**2**	0.03	**2**	0.03	**2**	**2**	**2**	**2**
*k_i_*	1.97	**4**	**4**	**4**	**4**	**4**	**4**	**4**	**4**
*k_e_*	0.44	**1**	0.44	**1**	0.44	**1**	0.44	**1**	**1**
*P_Cact_*	*	50	50	50	50	50	50	50	50
*k_Deg_*	1.36	1	**4**	**4**	1	1	1	1	1
*k_b_*	*	0.02	0.02	0.02	0.02	0.02	0.02	0.02	0.02
*Dl0*	*	36	**18**	**18**	36	36	36	36	36
*Dl-Cact0*	*	30	**15**	**15**	30	30	30	30	30
*Cact0*	36	36	36	36	36	36	36	36	36
Nuclear radius (*r*; µm)	3.08	3.08	3.08	3.08	**2.3**	**2.3**	**5.45**	**5.45**	**5.45**
DV nuclei (*n*)	100	92	92	92	**142**	**142**	**72**	**72**	**72**
time (*t*; min)	65	65	65	65	**55**	**55**	**86**	**86**	**86**

Revised parameter values discussed in the text are indicated in bold. For abbreviations of model parameters, see Table S2. *Dl0, Dl-Cact0* and *Cact0* indicate the cytoplasmic concentrations of Dl, Dl-Cact and Cact, respectively, at the onset of the final nuclear cycle. Time indicates the duration of the last nuclear cycle interphase (nc14 for wild type *melanogaster*). First column with “original parameters” lists one representative set of parameters from [15].

* Due to the non-dimensionalization, the original values of these parameters were unknown.

**Figure 4:**
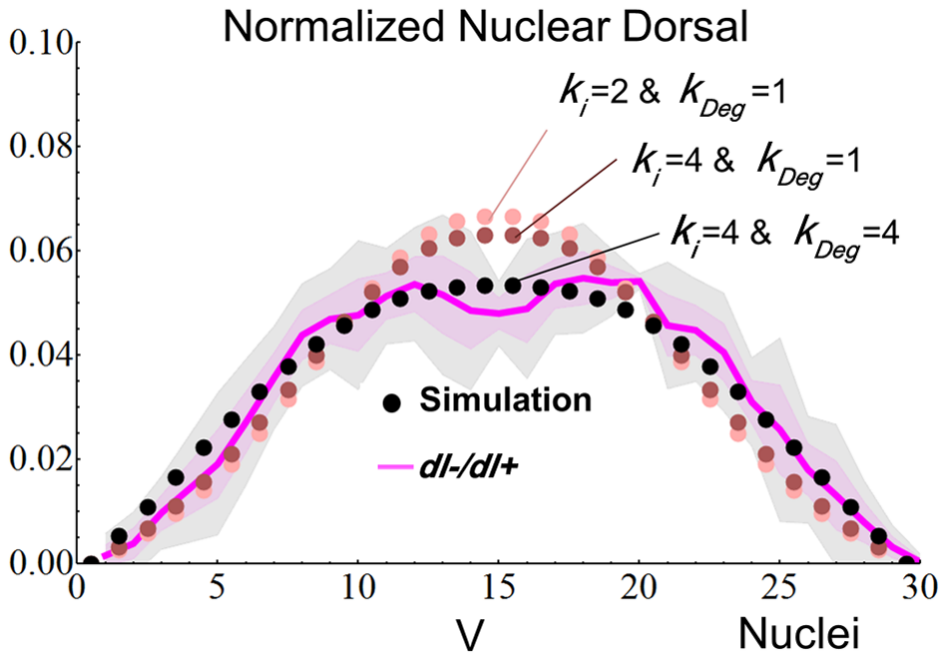
Changes in *k_Deg_* allow the reproduction of the Dl gradient in embryos derived from *dl^−^/dl^+^* mothers. Solid line and pink shadow, experimental quantification of Dl nuclear levels in the 30 most ventral nuclei (mean±SD, n = 5). Dotted lines indicate simulations with original values (light pink dots) for Dl nuclear import rates (*k_i_*) and Cact degradation (*k_Deg_*), and adjusted values (dark pink and black dots). The other parameters values used in the simulations are shown in the third column of Table 1. Gray shadow is a larger confidence interval (99%) due to the small sample size, and indicates that the slight deepening in the experimental gradient peak is not significant. **V** indicates ventral midline, *y* axis indicate normalized Dl levels, *x* axis indicate nuclei. See Table S5 for fit calculations.

The analysis of *dl−/dl+* embryos provided valuable insights about the model parameters. In agreement to a previous report [22], we verified that these mutants have a flattened Dl gradient (Fig. 4), which suggests that near the ventral midline, all cytoplasmic Dl is translocated into the nucleus. Therefore, it is reasonable to assume that in the wild type, the Dl nuclear import rate (*k_i_*) is not the limiting factor for the formation of the gradient peak. In other words, given enough Tl receptor activation and cytoplasmic Dl, peak levels of nuclear Dl can be achieved in the wild type. This result motivated us to increase the *k_i_* value (Table 1). We next asked if decreased Dl levels could simulate the *dl^−^/dl^+^* gradient shape. However, our model showed that the shape of the Dl gradient is insensitive to the initial concentrations of Dl, Cact and Dl-Cact (Fig. S5), unless these initial concentrations are zero, in which case the Dl gradient is not formed. This finding suggests that the gradient shape observed in *dl^−^/dl^+^* embryos is caused by additional parameter changes besides initial Dl concentration.

Several studies report that Cact is stabilized in the presence of Dl and that Cact levels are reduced if Dl levels are diminished [23]–[25]. Based on this information, we tested if changes in the rate of Cact degradation (*k_Deg_*) were able to reproduce the mutant gradient. We found that doubling the wild type *k_Deg_* value was not sufficient to completely reproduce the *dl−/dl+* flattened gradient. This finding suggests that the relationship between Dl amounts and Cact stabilization is not linear and probably involves cooperativity. Indeed, Dl is reported to form dimers, such that the Dl-Cact complex is formed by one unit of Cactus bound to two units of Dl [23], [25]. By increasing *k_Deg_* four times, our model could correctly reproduce the Dl gradient from *dl^−^/dl^+^* embryos (Fig. 4, Table 1).

**Figure 5:**
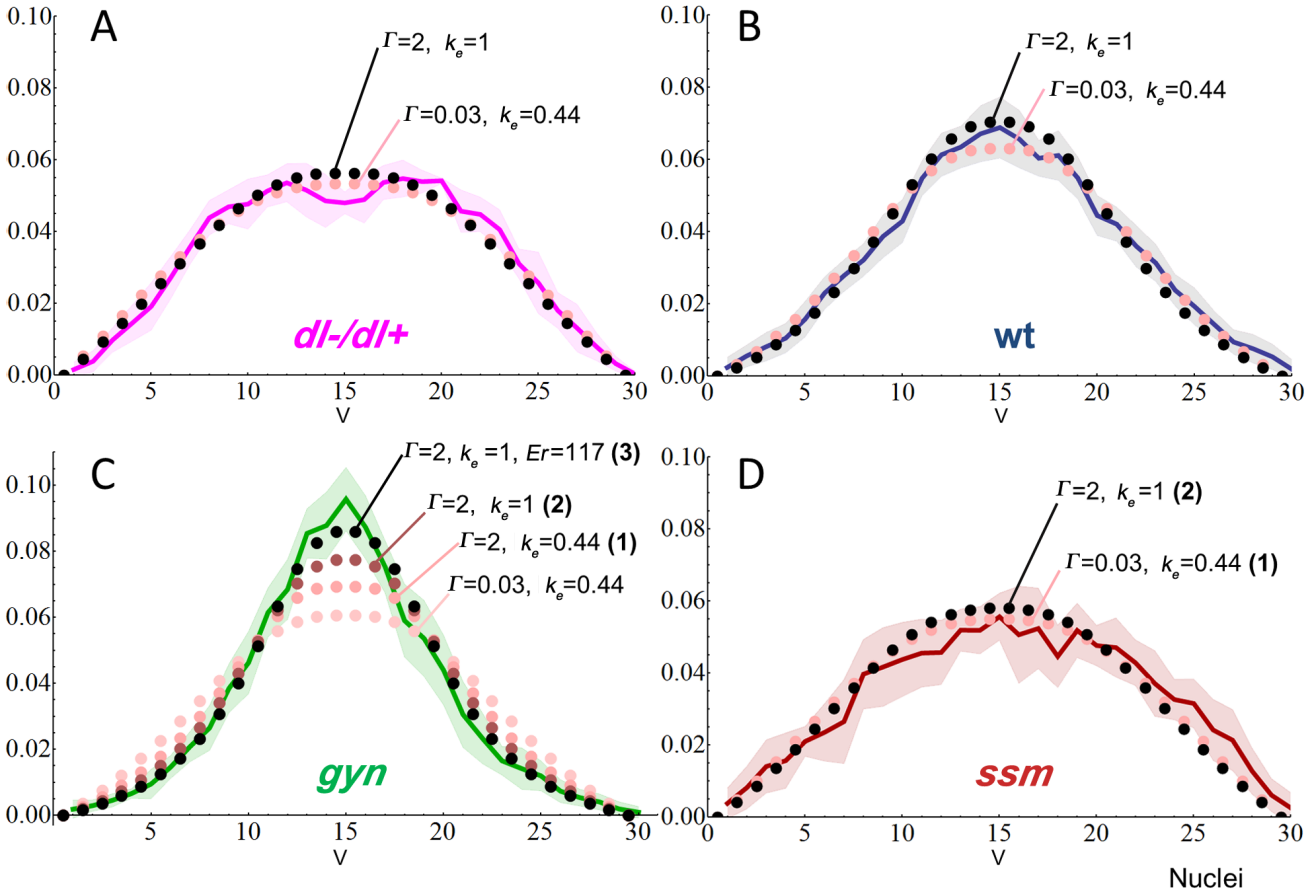
Simulations of wild type and mutant gradients suggest increased diffusion and Dl export rates. A–D) Comparison between experimental data (solid lines) and simulations (dotted lines) using parameter sets shown in Table 1. Simulations *gyn* 1–3 and *ssm* 1–2 from table are indicated by the number in parenthesis in (C) and (D), respectively. **V** indicates ventral midline, *y* axis indicate normalized Dl levels, *x* axis indicate nuclei. See Table S5 for fit calculations. Shaded areas represent average±SD.

After implementing this adjusted parameter set, our simulations still failed to reproduce the *gyn* gradient, unless three additional changes were made: (1) an increased diffusion rate among compartments, (2) an increased Dl nuclear export rates, and finally (3) an increased embryo radius (Fig. 5). Increasing Dl, Cact and Dl-Cact transport rates between adjacent compartments (*Γ*) from 0.03 to 2 sharpened the *gyn* gradient simulation (Fig. 5C, simulation 1) and still kept a good fit between the simulated and experimentally obtained gradients of wild type, *ssm*, and *dl−/dl+* embryos (Fig. 5A, B, D) (For fit calculations, see Table S5). Indeed, the fit was actually improved for the wild type (Fig. 5B, black dots; Table S5). The most common value of transport rate within the parameter vectors in the Kanodia model is 0.0064. We verified that the transport rate constant of 2 tested here falls within the parameter vectors found in the Kanodia model, albeit at low frequency [15]. By also increasing the Dl nuclear export rate (*k_e_*) from 0.44 to 1, the fit for *gyn* improved significantly (Fig. 5C, simulation 2) without compromising the wild type (Fig. 5B, black dots) and *ssm* simulations (Fig. 5D, black dots), and only having a small increase of the Dl peak levels in the *dl−/dl+* gradient simulation (Fig. 5A, black dots; Table S5).

In sum, increasing diffusion across compartments and Dl export rates greatly improved the *gyn* gradient simulation and did not impact significantly other *D. melanogaster* mutants and wild type simulations. Finally, an almost perfect fit for the *gyn* gradient was obtained by increasing the embryo radius (Fig. 5C, simulation 3). Although the main motivation to use *ssm* and *gyn* mutants to test the influence of nuclei size and density was the fact that these mutants should *a priori* have wild type egg sizes and a normal DV signaling pathway, actual measurements indicate that *gyn* has a slightly larger radius of 117 µm in comparison to 100 µm in the wild type.

The lack of a perfect simulation of the steep *gyn* gradient may be due to simplifications in the model. For instance, while Dl-Cact dissociation is the main response to Tl activation, the removal of Dl and Cact interaction is insufficient to promote maximum peak levels of nuclear Dl [23]. However, the model does not include alternative pathways for Dl nuclear translocation or possible interactions between Dl and other IkB related proteins. Also, the model does not represent other alternative DV polarizing sources involving components upstream of Toll, but the effect of this second polarizing signal is reported to be subtle and may not necessarily have measurable effects in a wild type background [26]. Together, our simulations nonetheless clearly indicate that embryo morphology affects the Dl gradient shape, and is likely to play an important role in the modifications seen in the other *Drosophila* species.

### Embryonic morphology alone does not fully explain species-specific Dl gradient shapes

**Figure 6:**
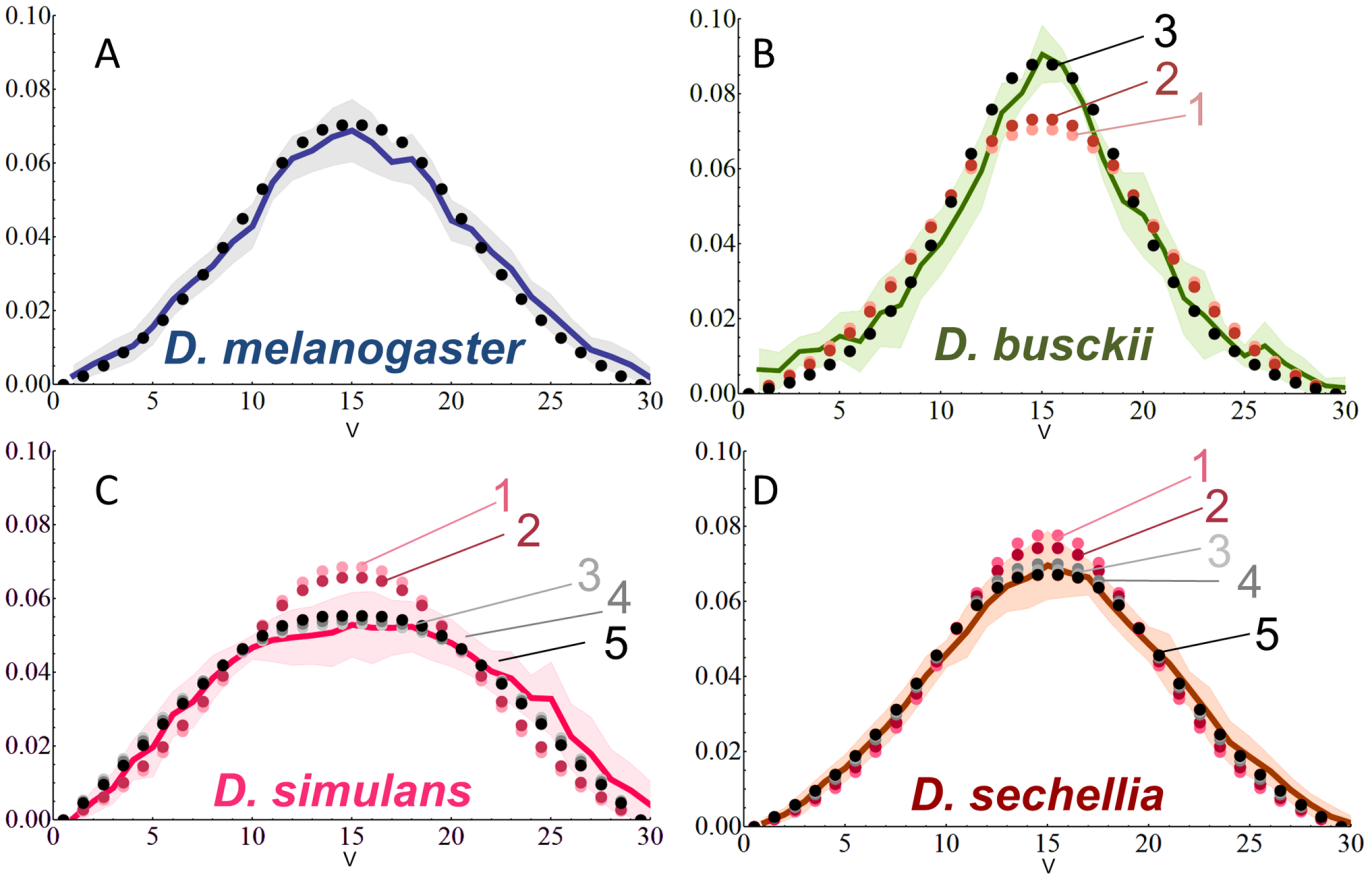
The model predicts similar adjustment in parameters consistent to the phylogenetic relationship of species. Experimental quantification of the Dl gradient (solid line) and model simulations (dotted lines). Shadowed area indicates mean±SD A–D) *D. melanogaster* (A, adjusted parameters shown in Table 1); and species gradient simulations according to Table 2 (B, *busckii;* C, *simulans*; D, *sechellia*). Experimental data was obtained from [8]. **V** indicates ventral midline, *y* axis indicate normalized Dl levels, *x* axis indicate nuclei. See Table S5 for fit calculations.

Since embryo geometry, nuclear density and nucleus radius affect the shape of the Dl gradient (Fig. S5), we first addressed how this restricted set of parameters act together to generate the species-specific Dl gradients we analyzed previously [8]. Embryos from *D. busckii*, *D. simulans* and *D. sechellia* have different sizes and geometries, as well as distinct nuclear density and size (Table 2). After adjusting these parameters to the values obtained experimentally, we verified that the model fails to reproduce the species-specific Dl gradients (Fig. 6, Table 2, simulations 1). Furthermore, additional simulations also discarded other parameters relative to morphology with no significant impact to the gradient shape; namely embryonic AP length, width of cortical layer and the total number of nuclei in the entire embryo (See Fig. S5 for simulations in *D. melanogaster*). We conclude that the evolutionary morphological modifications in these species alone are not sufficient to generate their final Dl gradient shape.

**Table 2:** Selected parameter sets used in model simulations of Dl gradient for three different *Drosophila* species, *D. busckii*, *D. simulans* and *D. sechellia*.

	bus 1	bus 2	bus 3	sim 1	sim 2	sim 3	sim 4	sim 5	sec 1	sec 2	sec 3	sec 4	sec 5
*El*	**189**	**189**	**189**	**236**	**236**	**236**	**236**	**236**	**286.5**	**286.5**	**286.5**	**286.5**	**286.5**
*Er*	**93.4**	**93.4**	**93.4**	**102.5**	**102.5**	**102.5**	**102.5**	**102.5**	**134.85**	**134.85**	**134.85**	**134.85**	**134.85**
*Eh*	**23.4**	**23.4**	**23.4**	**30.1**	**30.1**	**30.1**	**30.1**	**30.1**	**29.6**	**29.6**	**29.6**	**29.6**	**29.6**
*Tn*	6000	6000	6000	6000	6000	6000	6000	6000	**7000**	**7000**	**7000**	**7000**	**7000**
*R*	15000	**12142**	**12142**	15000	**19285**	**19285**	**19285**	**19285**	15000	**18571**	**18571**	**18571**	**18571**
*S*	4000	4000	4000	4000	4000	4000	4000	4000	4000	4000	4000	4000	4000
*ξ*	2.5	2.5	2.5	2.5	2.5	2.5	2.5	2.5	2.5	2.5	2.5	2.5	2.5
*Γ*	2	2	2	2	2	2	2	2	2	2	2	2	2
*k_i_*	4	4	4	4	4	4	4	4	4	4	4	4	4
*k_e_*	1	1	1	1	1	1	1	**0.5**	1	1	1	1	**0.5**
*P_Cact_*	50	50	50	50	50	50	50	50	50	50	50	50	50
*k_Deg_*	1	1	**0.33**	1	1	**5**	1	**2**	1	1	**1.5**	**1.5**	1
*k_b_*	0.02	0.02	0.02	0.02	0.02	0.02	**0.005**	**0.015**	0.02	0.02	0.02	**0.015**	**0.015**
*Dl0*	36	36	36	36	36	36	36	36	36	36	36	36	36
*Dl-Cact0*	30	30	30	30	30	30	30	30	30	30	30	30	30
*Cact0*	36	36	36	36	36	36	36	36	36	36	36	36	36
*r*	**2**	**2**	**2**	**2.75**	**2.75**	**2.75**	**2.75**	**2.75**	**3.5**	**3.5**	**3.5**	**3.5**	**3.5**
*n*	**86**	**86**	**86**	**98**	**98**	**98**	**98**	**98**	**102**	**102**	**102**	**102**	**102**
*t*	**65**	**65**	**65**	**65**	**65**	**65**	**65**	**65**	**65**	**65**	**65**	**65**	**65**

Bold numbers indicate modified values in relation to *D. melanogaster*. Abbreviations: **bus**, *D.* busckii; **sim**, *D. simulans*; **sec**, *D. sechellia.* For abbreviations of model parameters, see Table 1 and Table S2.

### Modulating a small subset of parameters affecting the Toll signaling pathway can reproduce species-specific Dl gradients

We reasoned that the next logical step requiring minimal model manipulations to achieve good gradient fits for the species should involve adjusting parameters that regulate the Tl signaling pathway. This idea is supported by the fact that the Tl pathway is a fast-evolving pathway in Drosophilids, which is required for immune response in addition to DV patterning [27]–[31] (See Text S5 for selection of parameters). Furthermore, we previously showed that this pathway is indeed modified in the species, as seen by their distinct ranges of peak Tl activation levels measured as the percentage of arc-length occupied by the mesodermal marker *sna* [8]. This variation goes from 21% in *D. melanogaster* to 17% in *D. busckii*, 26% in *D. sechellia* and 27% in *D. simulans*.

We tested the effect of three parameters (*R*, *S* and *ξ*) that influence the amplitude and shape of the space-dependent Dl-Cact dissociation rate constant (*k_D_*) and as such control the range of Tl signaling strength extending dorsally from the midline. By modifying either *R* or *ξ*, we could obtain simulations with good fit for each species. For instance, *D. sechellia* gradient can be reproduced using an *R* value of 50,000, but *D. simulans* requires a much larger value of 114,000. However, such large difference in *R* values is not supported by the experimental data showing these two species have nearly identical mesodermal percent arc-length [8].

Assuming a linear relationship between the percent arc-length of the mesoderm and *R,* we tested adjusted *R* values of 12,142 (*busckii*), 19,285 (*simulans*) and 18,571 (*sechellia*). These more modest changes in *R* slightly improve all simulations (Fig. 6, simulations 2; Table 2). Most importantly, the gradients are correctly reproduced by few additional changes in Tl pathway parameters, and these changes agree with the phylogenetic relationship of these species. For instance, in the two most closely related species *D. simulans* and *D. sechellia*, either increasing Cact degration rates (*k_Deg_*) or reducing Cact production rates (*P_Cact_*) can correctly simulate their gradients (Fig. 6C, D, simulations 3; Table 2). In other words, the significantly different gradients observed in these species, which vary in nuclear and embryo size, are generated by changes in the same parameters and place them apart from *D. melanogaster*. In contrast, the model predicts that *D. busckii*, a more distantly related species from the *melanogaster* subgroup, requires an opposite change over Cact regulation, i.e., a decrease in *k_Deg_* or increase in *P_Cact_* in order to simulate its gradient (Fig. 6B, simulation 3; Table 2).

To gain further insights about the more closely related species *D. simulans* and *D. sechellia,* we tested additional parameters that regulate Dl and Cact functions. We found that decreasing binding of Cact to Dl (*k_b_*) also generates a good fit for these two species (Fig. 6C, D, simulations 4; Table 2). Another prediction made by the model was that decreasing Dl export rates in both *D. simulans* and *D. sechellia* can also improve the simulation of their gradients (Fig. S6). Finally, by simultaneously modifying more than two Tl-related parameters at a time, we also obtained good fits for *D. simulans* and *D. sechellia* (Fig. 6C, D, simulations 5; Table 2). We also observe the same overall model behavior when using various randomly generated parameter sets within the range of the parameter cloud identified in the original model [15] (Figure S7, Table S6). These results indicate that the very distinct Dl gradient shapes found in these closely related species can be correctly simulated by making similar modifications in selected parameters involved in Tl pathway.

### Analyses of another pair of closely related sibling species suggest evolutionarily shared mechanisms for Dl gradient formation

As seen above, our simulations indicate that making similar adjustments in parameters that affect Cact regulation or Dl export rates generate good fits for *D. simulans* and *D. sechellia*. We expected that the model could reveal if there were common evolutionary mechanisms for the formation of the Dl gradient in another pair of sibling species, *D. santomea* and *D. yakuba,* which would also set them apart from *D. melanogaster*. *D. santomea* emerged as recently as *D. sechellia* (Fig. 1C) and is also reported to have enlarged egg size [6], but the speciation of these two species took place in geographically distinct regions [14].

We obtained measurements of embryo size, nuclear size and density for these species (Table 3). Dl quantifications in both *D. yakuba* and *D. santomea* reveal an overall gradient shape similar to *D. melanogaster* and *D. sechellia,* except for slightly lower peak levels in *D. yakuba.* Interestingly, the percent arc-length of *sna* in *D. yakuba* and *D. santomea* (22.06%, SD = 1.92, n = 5; and 20.44%, SD = 1.54, n = 5, respectively) is similar to *D. melanogaster*, suggesting that the broadening of Tl range is an innovation in the branch of *D. simulans* and *D. sechellia*.

**Table 3:** Selected parameter sets used in model simulations of Dl gradient for *Drosophila yakuba* and *Drosophila santomea*.

	yak 1	yak 2	yak 3	yak 4	san 1	san 2	san 3	san 4
*El*	**251.6**	**251.6**	**251.6**	**251.6**	**302.45**	**302.45**	**302.45**	**302.45**
*Er*	**114**	**114**	**114**	**114**	**126**	**126**	**126**	**126**
*Eh*	**24.85**	**24.85**	**24.85**	**24.85**	**32.43**	**32.43**	**32.43**	**32.43**
*Tn*	6000	6000	6000	6000	6000	6000	6000	6000
*R*	15000	**16578**	**16578**	**16578**	15000	**15789**	**15789**	**15789**
*S*	4000	4000	4000	4000	4000	4000	4000	4000
*ξ*	2.5	2.5	2.5	2.5	2.5	2.5	2.5	2.5
*Γ*	2	2	2	2	2	2	2	2
*k_i_*	4	4	4	4	4	4	4	4
*k_e_*	1	1	1	1	1	1	1	1
*P_Cact_*	50	50	50	50	50	50	50	50
*k_Deg_*	1	1	1	**2**	1	1	1	**2**
*k_b_*	0.02	0.02	**0.01**	0.02	0.02	0.02	**0.01**	0.02
*Dl0*	36	36	36	36	36	36	36	36
*Dl-Cact0*	30	30	30	30	30	30	30	30
*Cact0*	36	36	36	36	36	36	36	36
*r*	**3.4**	**3.4**	**3.4**	**3.4**	**3.7**	**3.7**	**3.7**	**3.7**
*n*	**100**	**100**	**100**	**100**	**96**	**96**	**96**	**96**
*t*	65	65	65	65	65	65	65	65

**Figure 7:**
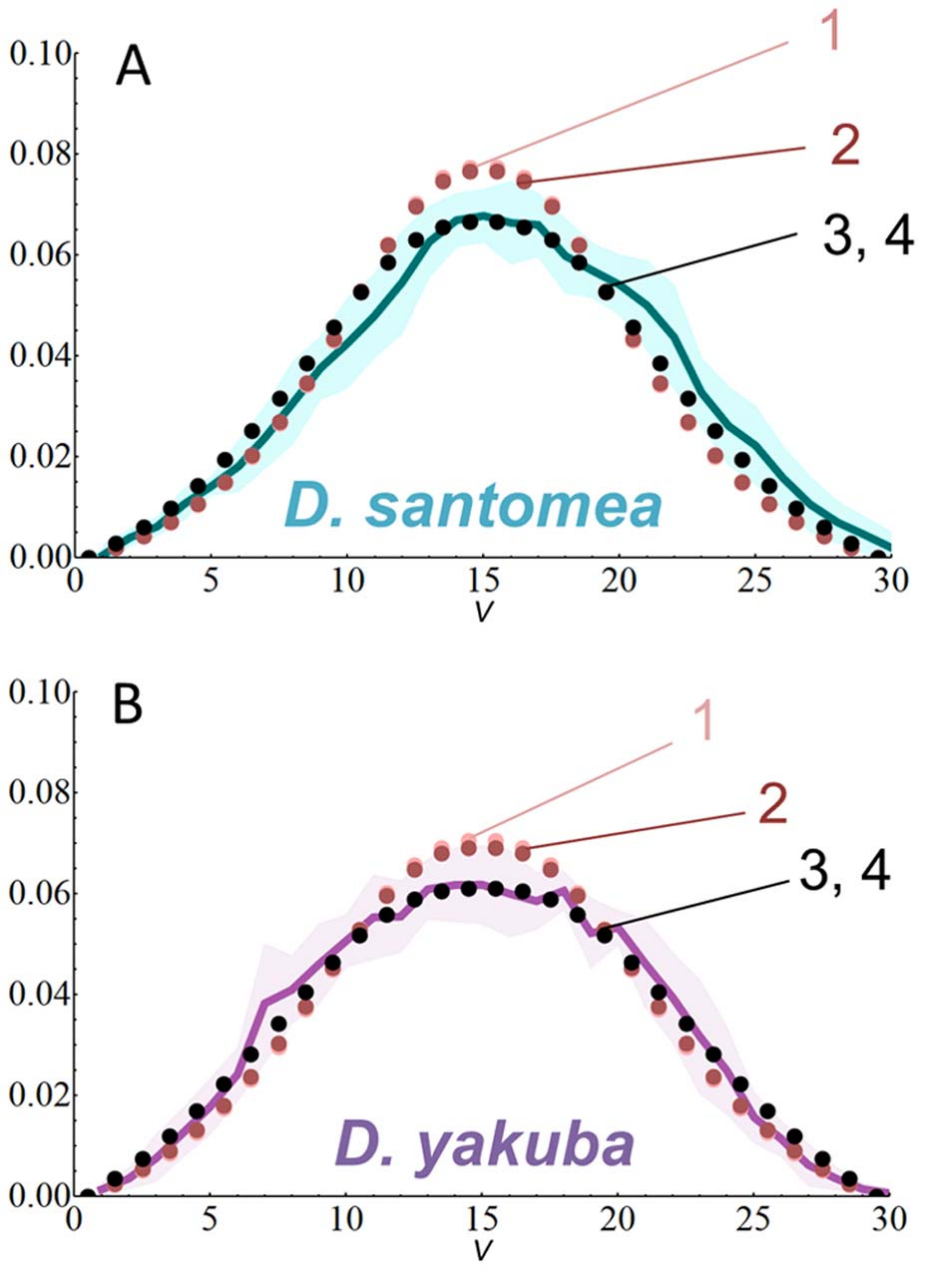
Simulations of Dl gradient in an additional pair of closely related species with varying egg size suggest a shared Dl-Cact binding rates or Cact degradation rates. Experimental quantifications (solid lines) and simulations (dotted lines) in *D. santomea* (A) and *D. yakuba* (B) based on parameters indicated in Table 3. Shadowed area indicates average±SD. Best fitting curves are obtained with the same lowered *k_b_* or the same increased *k_Deg_* value for both species (black dots, simulation 3 and 4 respectively). **V** indicates ventral midline, *y* axis indicate normalized Dl levels, *x* axis indicate nuclei. See Table S5 for fit calculations.

Bold numbers indicate modified values in relation to *D. melanogaster*. Simulations 3 and 4 (shown in Fig. 7) provide the best fit. Abbreviations: **san**, *D. santomea*; **yak**, *D. yakuba.* For abbreviations of model parameters, see Table 1 and Table S2.

After adjusting the model parameters with the *D. yakuba* and *D. santomea* measurements of embryo, nuclear size and density, the resulting gradients were sharper than the experimentally measured gradients (Fig. 7, simulations 1). We were able to correctly simulate their gradients by modifying parameters related to the Tl pathway, such as decreasing *k_b_*, or increasing *k_Deg_* to a same value in both species (Fig. 7, simulations 3 and 4; Table 3). Decreasing Dl export rates also improves the simulations, but a comparison of Dl protein sequence did not indicate modifications in the Nuclear Export Sequences (NES) from *D. melanogaster* (see below). In sum, our model indicates that in the *D. yakuba* and *D. santomea* lineages, the Dl gradient formation appears to depend on similar modifications in Cact regulation, setting these species apart from *D. melanogaster* as was the case for *D. simulans* and *D. sechellia*.

### Dl and Cact protein sequence comparisons of *melanogaster* subgroup species support predictions made by the model

To further investigate the biological relevance of Cact regulation and Dl export rates in the formation of the species-specific Dl gradients, we analyzed the amino acid sequences of these proteins from the *melanogaster* subgroup species. We aligned *D. melanogaster* Dl with *D. simulans* and *D. sechellia* Dl sequences and found that all known functional domains of the protein are conserved, with the exception of the nuclear export sequence 3 (NES3), which contains 3 amino acid (aa) substitutions in *D. simulans* and *D. sechellia* (Fig. S8A). These changes could potentially decrease Dl export rates in these species [32], [33], as predicted by our model. In contrast, *D. yakuba* and *D. santomea* exhibit identical sequences of all NES domains to *D. melanogaster.*

Although the model equations do not capture the full complexity of the Cact degradation pathways *in vivo*, the comparison of Cact sequences from these species also provided further support for possible changes in its regulation (Fig. S8B). The Cact C-terminal contains six ankyrin repeats [34] which are necessary for its binding to Dl. We found that *D. simulans* and *D. sechellia* contain an insertion of 15 aa within the beginning of ankyrin repeat 4. Using Phyre2 software to predict protein structure [35], we verified that this insertion does not eliminate this ankyrin motif itself but it may create two α-helixes between ankyrin repeats 3 and 4, in contrast to only one long helix present in *D. melanogaster* Cact. Likewise, *D. yakuba* Cact is also predicted to have two α-helixes in the same region, due to some nearby aa substitutions. It is possible that the alteration nearby the ankyrin domains could modify the binding between Dl and Cact in these species, which would further support the model prediction that using a lower *k_b_* rate than *D. melanogaster* yield good fits of the other species simulations.

Another important regulatory region in Cact sequence is located in the N-terminal (Fig. S8B). This region is rich in serine residues that are phosphorylated in response to Tl activation, leading to Cact degradation. *D. simulans* contains only one serine substitution (S94R) in relation to *D. melanogaster*, but this site has never been tested for its function *in vivo. D. yakuba* contains more Cact modifications in relation to *D. melanogaster,* with a total of 18 aa substitutions, including 4 serine substitutions. In addition, *D. yakuba* Cact has a deletion of 9 aa at positions 124–132, nearby a domain previously implicated in Cact degradation *in vivo* [36]. Together, these variations in Cact and Dl suggest that subtle and additive, but possibly biologically relevant changes in components of the Tl pathway are shared by the most closely related species and may contribute to their final Dl gradient shape, as suggested by our model simulations.

### Model robustness and sensitivity analysis reveals non-linear interactions between species morphology modifications and other relevant parameters

**Figure 8:**
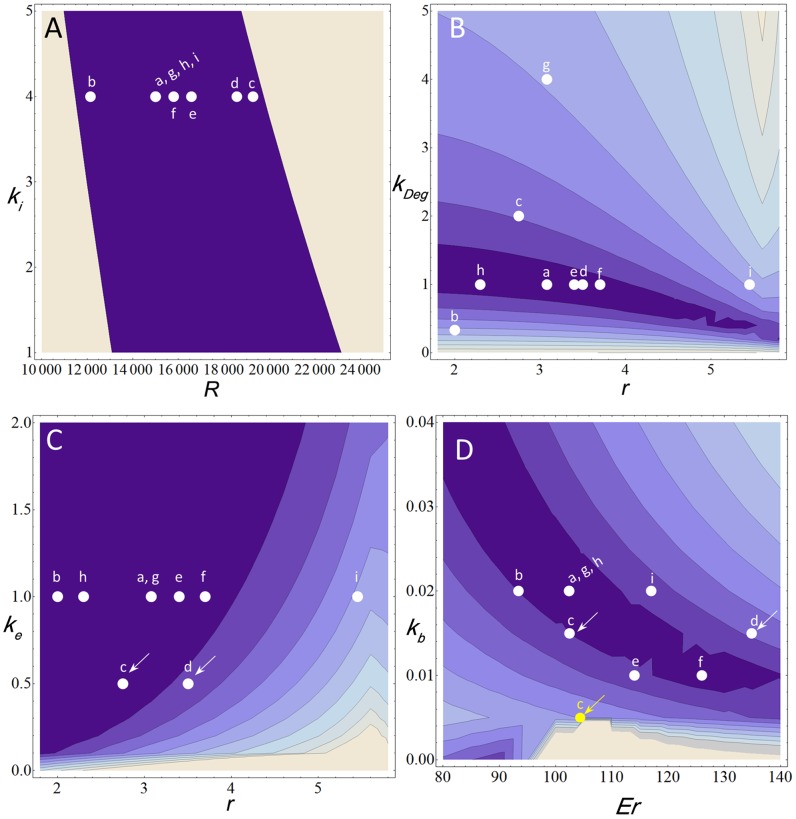
Sensitivity analysis for main parameters tested. A–D) Contours represent a drop of 0.01 in fit (square root of the sum of square differences between the gradient produced with the parameter values showed in the *y* and *x* axis and the gradient produced with wild type *melanogaster* parameters), with exception to (A), in which the drop in fit is 0.0094. A) Example of all simulations within highly robust regions of the model (*k_i_* x *R*). B) Example of *gyn* (“i”) and *D. busckii* simulations (“b”) that fall within less stable regions of the model compared to other samples. C) Lowering Dl export rates (*k_e_*) in *D. sechellia* (“d, arrow”) and *D. simulans* (“c, arrow”) allows good fits for these species after correcting for nucleus radius (*r*). D) Simulations of *D. simulans* that rely on drastic changes in only one parameter, in this case, *k_b_*, fall within unstable regions of the model (compare “c” white and yellow dots indicated by arrows.) **a**, *D. melanogaster*; **b**, *D. busckii;* **c**, *D. simulans,* **d**, *D. sechellia;* **e**, *D. yakuba;* **f**, *D. santomea;* **g**, *dl−/dl+;* **h**, *ssm*; **i**, *gyn*.

We next carried out a sensitivity analysis to test how robust the model is to simultaneous changes in the relevant parameters identified above. Instead of an exhaustive test for all possible combinations of parameter values, we focused on the effects on the model output when changing only two concomitant parameters at a time. We observe that for most combinations tested, the simulations stay within robust regions of the model (Fig. 8A; Fig. S9 K, V–X). Two simulations in particular tend to fall within slightly more unstable regions of the parameter range, namely *gyn* and *D. busckii* (Fig. 8B, see also Fig. S9E, L–Q, S, U).

Our analysis confirmed that the model is indeed sensitive to changes in two key parameters for reproducing the species gradients, Cact degradation (*k_Deg_*) and Dl-Cact binding rates (*k_b_*) (Fig. 8B, D). Furthermore, we observe non-linear interactions between *k_Deg_* and *k_b_* and parameters related to embryonic morphology (Fig. 8B, D). For instance, in *D. simulans* and *D. sechellia*, changes in Dl-Cactus binding rates (*k_b_*) affect the Dl gradient distribution outcome caused by changes in embryonic radius (*Er*; Fig. 8D) and nuclear radius (*r*; Fig. S9J). In addition, while the model is mostly robust to changes in nuclear Dl export rates (*k_e_*; Fig. 8C, Fig. S9R, T), it does display more sensitivity to *k_e_* when paired with the species-specific nuclear radius (Fig. 8C) and embryo radius variations (Fig. S9Q). Together, these results support an overall robustness of the model simulations and reveal an interaction between morphological modifications and the few selected parameters of Tl pathway regulation that improve the species-specific simulations.

## Discussion

Variation in species size creates a challenge on how gene expression patterns can accommodate to new embryonic dimensions without compromising cell fates and viability. Our study of DV patterning response to physical and biochemical changes in mutants and *Drosophila* species provided new insights on Dl gradient scaling. First, the model indicates that changes in parameters such as embryo size and nuclear size, which are commonly found in several *Drosophila* species, are not sufficient to recreate the Dl gradient shapes seen in these species. However, these parameters interact with a small subset of parameters related to Tl pathway, which when modified, are sufficient to generate simulations with good fits with the experimental Dl gradients. Our results also suggest that those changes in Tl pathway are likely to have been shared within closely related lineage branches, which is further supported by the sequence comparisons of Dl and Cact proteins from these species. Thus, the mathematical modeling used here advances our understanding on how gradient shapes are acquired during evolution, which could not be explained by solely quantifying and comparing Dl levels across species.

### Dorsal scaling within and across species

Garcia *et al.* [10] recently investigated the Dl gradient scaling within the same species using *D. melanogaster* lines artificially selected to have small or large embryos [9]. Their study indicates that the Dl gradient width is positively correlated with DV axis length and the number of nuclei along the DV axis. Our experimental data from ploidy mutants and mathematical simulations support the claim that an increase in the number of DV nuclei causes a widening of the Dl gradient. Garcia *et al.* [10] also suggest that changes in the range of Tl signaling could explain the observed scaling of the Dl gradient width within *D. melanogaster* species. We previously found variations in the range of peak Tl signaling across species [8], and in this work we provide evidence for species-specific changes within the Tl signaling pathway as a means of influencing the Dl gradient shape.

We also show that increasing Dl nuclear export rate and diffusion between cellular compartments more accurately recreates *D. melanogaster* wild type and mutant Dl gradients (Fig. 5). With regard to diffusion rates, the majority of parameter sets found in the Kanodia model is in agreement with a cell autonomous steady state behavior, which is supported by live-imaging experiments showing that a GFP-tagged version of Dl has limited diffusion between neighboring compartments [37]. We verified that our adjusted diffusion rates do not exclude the possibility that the embryo is fully compartmentalized, but we also observe that the final Dl gradient shape is influenced by a non-cell-autonomous process (Text S4). Future work testing native Dl diffusion without GFP may resolve whether the Dl gradient formation is a non-cell-autonomous process with increased lateral diffusion that may be required for scaling the final gradient shapes observed in nature. The difference in embryo morphology across species is also expected to either increase or decrease the diffusion of Dl by itself, as it has been shown before in experiments that measured diffusion constants of injected dextran in species with small and large embryos [3]. These experiments revealed a trend of increased dextran diffusion in large embryos versus decreased diffusion in small embryos. Consistent with this finding, we also note that our calculated diffusion coefficient of Dl, Cact and Dl-Cact slightly increases in larger embryos (e.g. *D. sechellia* and *D. santomea*) and decreases in smaller embryos (e.g. *D. busckii*), after doing a unit conversion of *Г* that inputs the measured values for embryo morphology (Text S4).

Prior work showed a scaling of the antero-posterior gradient Bcd in the inbred *D. melanogaster* lines mentioned above [11], [12] and proposed a mechanism in which more maternal *bcd* mRNA is loaded into larger embryos to compensate for their increased size. With respect to the Dl gradient, an increase in nuclear Dl concentration can occur with or without a corresponding increase in embryo size or altering the maternal contribution of Dl. For instance, we found that *D. sechellia* and *D. santomea* do have greater concentrations of Dl in ventral nuclei in relation to their smaller sibling species *D. simulans, D. melanogaster* and *D. yakuba* [8] (Fig. S10). However, despite the fact that *D. simulans* produces embryos of comparable size to *D. melanogaster*, the nuclear Dl concentration levels in the former species are more elevated [8]. We show that changes in nuclear size and density, range of peak Tl activation and changes within the Tl signaling pathway provide additional strategies to altering nuclear Dl concentrations and distributions, which can work in conjunction with altering the maternal dosage of Dl.

### Model sensitivity analysis support evolution of Dl gradient by small additive changes in Tl regulation pathway

Two interesting properties of this system emerged from our robustness and sensitivity analysis. First, it can be seen that lowering Dl nuclear export rates for *D. simulans* and *D. sechellia* allows the model output to change from a flat to a sharp gradient shape after correcting for the species-specific nuclear radius (Fig. 8C, white arrows). A similar non-linear interaction is observed between Dl-Cact binding constant (*k_b_*) and nuclear radius (Fig. S8J, “c” and “d” points). Second, we notice that for *D. simulans* and *D. sechellia*, the simulations stay within robust regions when more than two parameters are modified at a time (e.g. *k_e_*, *k_Deg_* and *k_b_*, Table 2, simulations 5). In contrast, simulations that sharply decrease only one parameter at a time in *D. simulans*, such as decreasing Dl-Cact binding rates (*k_b_*) (e.g. Table 2, simulation 4) fall within more unstable regions (Fig. 8D, yellow arrow; see also Fig. S8F–J, yellow dots). In the case of *D. busckii*, we also note that the simulations fall within more unstable regions of the model upon changes in the parameter of Cact degradation (*k_Deg_*) only. These results suggest that it is unlikely that these species acquired their Dl gradient shapes by drastic regulatory changes that affect only one component of the Tl pathway.

### The Dl gradient model predicts changes in the Tl pathway in *Drosophila* species that are consistent to their phylogenetic relationships

The results obtained from the use of computational modeling revealed important properties about the behavior of gradient formation and evolution of the Tl signaling pathway in the *Drosophila* species tested. First, our analyses of *ssm* and *gyn* mutants demonstrate that the rapid changes in embryo size, nuclear size and density of these species can modify the Dl gradient shape, but those changes alone are not sufficient for the final species-specific Dl gradient shapes. The second significant prediction made by the modeling is that additional changes in the Tl pathway regulation are required for obtaining good fits with the experimental gradient shapes in these species.

At first, it was surprising to find that the gradients of the most distantly related species (i.e. *D. sechellia* and *D. melanogaster*) have an identical distribution, whereas the gradients of the closest related species *D. simulans* and *D. sechellia* acquired completely different shapes. However, predictions made by our model reconcile the fact that these quite different gradient shapes can in fact be generated by a similar dynamics of Tl signaling in *D. simulans* and *D. sechellia*, after adjusting for their divergent anatomy. First, as suggested in our previous work, the range of peak Tl activation is broader in *D. simulans* and *D. sechellia*, compared to *D. melanogaster.* Second, our present data suggest that additional modifications in components of the Tl pathway affecting Cact regulation and Dl export rates also diverged in the newest species. By altering these parameter to similarly higher (e.g. increased *k_Deg_*) or lower values (e.g. decreased *k_e_* or *k_b_*), good fits of the gradients are generated for both *D. simulans* and *D. sechellia* (Fig. 6, Table 2, and Table S5). In support of these findings, we verified that *D. simulans* and *D. sechellia* share similar changes in amino acid sequences of Cact and Dl within or near domains previously implicated in Dl-Cact binding and Dl nuclear export, respectively (Fig. S8).

The simulations of Dl gradients in another closely related pair, *D. yakuba* and *D. santomea*, also suggested shared modifications in Cact regulation. Either lowering Dl-Cact binding rates (*k_b_*) or increasing Cact degradation (*k_Deg_*) to same values can generate good fits for the gradients of these species (Fig. 7, Table 3). Genomic data available for *D. yakuba* confirmed the prediction that Cact sequences within domains involved in degradation and Dl binding are indeed modified in relation to *D. melanogaster.* In contrast, the Dl protein domains in *D. yakuba* are well conserved in relation to *D. melanogaster.* We partially sequenced Dl from *D. santomea* and found that these domains are similar to *D. yakuba*.

In sum, despite the fact that *melanogaster* subgroup species have particular egg sizes, nuclear size and density, and their Dl gradient shapes appear at odds with their phylogenetic relationships, the use of mathematical modeling reveals that most closely related species share similarly modified regulation of the Tl pathway inherited from their common ancestor.

## Materials and Methods

### Fly stocks

*yw D. melanogaster* was used as wild type. Haploid and triploid embryos were generated in our previous work [8] using the mutations *sesame* (*ssm*) [20] and *gynogenetic-2; gynogenetic-3* (*gyn*) [21]. The *D. busckii*, *D. sechellia* and *D. simulans* strains used in [8] were obtained from the *Drosophila* Species Center at UCSD. The *D. yakuba* (tai 6 line) and *D. santomea* (CAR 1495.5 line) stocks were obtained from Daniel Matute (Univ. of Chicago).

### Gradient quantification and measurements of nuclear size

Quantification of Dorsal gradient and normalization method are described in detail in [8]. Briefly, embryos were stained for anti-Dorsal antibody (Iowa Hybridoma Bank) and a Donkey anti-mouse Alexa 647, manually sliced in cross-sections within trunk region and imaged using a LSM700 Zeiss Confocal microscope. Fluorescent intensity from the 30-most ventral nuclei was obtained using Axiovision software (Zeiss). Position of midline was estimated with a double staining for *snail* RNA. For nuclei diameter measurement, early-stage embryos stained with anti-Laminin (Iowa Hybridoma Bank) were mounted longitudinally with glass beads (150–210 µm size, Polysciences), to prevent flattening caused by the coverslip. Confocal slices were taken from the embryo surface to its mid-section and nuclei diameter was determined using ImageJ software. In the case of *ssm* and *gyn* mutations, some additional measurements were taken from embryos stained with DAPI nuclear dye.

### Reproduction and modification of the Kanodia model in Mathematica

The nondimensionalized model of nc10–14 was reproduced as described by Kanodia *et al.* [15]. Simulations of *gyn* and *ssm* gradients employed same equations (Text S1), with the following genotype-specific changes in the parameter values. Nuclei radius and density along the DV axis at the last nuclear cycle were directly measured as described above and in [8]. Total embryonic nuclei density at final cyles in *ssm* (nc15) and *gyn* (nc13) were estimated at 1200 and 3000, respectively, based on the fact that wild type embryos have an estimated 6000 nuclei at nc14 and on previous data for haploid embryos [38]. The number of nuclei along the DV axis (*n*) at early cycles was obtained as in Kanodia *et al.* [15] by multiplying *n* by 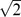 after each cycle. In our modified model, we adjusted the final number of DV nuclei at nc14 for *D. melanogaster* wild type from 100 to 92, as experimentally obtained in [8]. Adjustments for nuclei size in early nuclear cycles and developmental timing in *ssm* and *gyn* embryos are explained in Text S2 (see also Table S4 and Fig. S4), and were estimated based on [39]–[41]. Parameter changes for other *Drosophila* species were done according to data measured here, and in previous work [8], [15], [42] as described in the main text.

### Dimensionalized model of the last nuclear cycle

Dimensionalized equations were written in Mathematica using original mass-balance equations from the Kanodia model (Table S1). Additionally, to better represent the changes in embryo volume between species, instead of linearizing the cellular compartments as in the original model, those were represented as circular trapezoids organized in a circle (Fig. S11). The whole cross-section was modeled, with no need for no-flux boundary conditions. Details of the modifications are described in Text S3.

### Model validation

The original Kanodia model was validated here against three mutant conditions within the same species *D. melanogaster* (*dl−/dl+, ssm, gyn*). Manual adjustments in the parameter *k_i_* was made for *dl−/dl+,* and adjustments of *Γ, k_e_* were made for *gyn* (See main text). Those same values were maintained for a second round of simulations for wild type, *ssm* and *dl−/dl+*, which served as internal validation controls. Fit between experimental and simulation graphs remained roughly similar for *ssm* and *dl−/dl+*, and it was improved for wild type (Table S5).

### Fit calculation and confidence intervals

For fitness comparison, the square root of the square differences between the simulated gradients and respective experimental data was calculated and provided in Table S5. Standard deviation of the mean (SD) are indicated by error bars (Fig. 2) or shadowed areas (Fig. 4–7). Pink shadowed area in Fig. 3 (*dl* ^−^/*dl* ^+^ mutants) indicates SD, gray shadow indicates the 99% confidence interval for the experimental mean, as explained in the figure legend. Simulations “1” for *D. busckii*, *D. simulans* and *D. sechellia* (Fig. 6) lie outside of the 99% confidence interval (not shown), and are statistically different from best fit simulations (black dots). Simulations “1” for *D. yakuba* and *D. santomea* (Fig. 7) lie within the 99% confidence interval. However, even though there is no statistical significance, simulations “3” and “4” have an improved fit according to our fit calculations.

### Sequence comparison of Dl and Cact in *melanogaster* subgroup species

Available coding sequences of Dl and Cact for *D. simulans, D. sechellia* and *D. melanogaster* were obtained from FlyBase and aligned using tblastn (NCBI). For *D. santomea*, genomic DNA was amplified, sequenced and analyzed as described above. Fig. S8 summarizes the comparison for the sequences obtained. Protein structure analysis was done using Phyre2 software (http://www.sbg.bio.ic.ac.uk/phyre2/html/page.cgi?id=index). Location of Cact and Dl conserved domains was based on previous work [32], [33], [36], [43], [44].

## Supporting Information

Figure S1 Reproduction of the original simulations from Kanodia *et al*. [15] in Mathematica. (A) 3D plots of wild type Dl gradient from nuclear cycle 10 to 14 (nc10–nc14). (B) Amplitude of the Dl gradient at the end of each interphase (see color code). (C) Dl gradient at the end of each interphase (same color code) normalized as a percentage of the highest nuclear Dl level, highlighting that the shape of the Dl gradient is conserved throughout development. Figures A, B and C should be compared to Figure 4A, 4B and 5D(ii), respectively, from Kanodia *et al*. original publication [15]. V: ventral midline; D: dorsal midline.
(TIF)

Figure S2 Simulations of changes in nuclear radius and the effect over Dl gradient shape. (A–C) Changes in nuclear radius (*r*) at nc10–13 affect the shape of the gradient at the respective cycles, but not at the last nuclear cycle. (D) Superposition of the gradients at the end of the last nuclear cycle from A–C (green arrows). V: ventral midline; D: dorsal midline.
(TIF)

Figure S3 Plotting the Dl gradient along the entire DV axis or against percent mesoderm width reveal similar Dl distributions. (A) Wild type and (B) *ssm* blastoderm cross-sections stained for Dl protein (grey). Border of the 30 most ventral nuclei is indicated in one of the embryo sides (arrow, left side). Dorsal nuclei beyond this border have no detectable Dl signal with our method employed and the ratio of noise to signal is very high. (C) Normalization of the Dl gradient along the 30 most ventral nuclei for wild type (blue) and *ssm* (red), as used in this paper. (D) Dl gradient plotted against percent mesoderm width, where 19 nuclei comprise 100% mesoderm width in wild type *D. melanogaster* (blue) and 25 nuclei comprise 100% mesoderm width in *ssm D. melanogaster* (red). Note that differences in Dl distribution between wild type and *ssm* are clearly discernible (C, D).
(TIF)

Figure S4 Time-dependent nuclear radius dynamics for wild type (blue), *ssm* (red) and *gyn* (green) used in Figure 3 and Figure S2.
(TIF)

Figure S5 Individual influence of 19 parameters on the final shape of the Dl gradient. All graphs represent normalized nuclear Dl levels for the 30 most ventral cells of a cross-section at the end of nuclear cycle 14. With the exception of the parameter being manipulated, the values of the parameters used for all simulations are shown in Table 1, under *D. melanogaster* adjusted parameters. Note that the last two graphs show the effect of duration of interphase with distinct values of transport rates between compartments (*Г*).
(TIF)

Figure S6 Simulations with changes in Dl nuclear export rates (*k_e_*) only in *D. simulans* and *D. sechellia.* (A–D) Experimental quantification of the Dl gradient (solid line; shadow represents average±SD) and model simulations (dotted lines) for *D. simulans* (A, C) and *D. sechellia* (B, D). Dark pink dotted lines represent simulations “sim 2” and “sec 2” (Table 2). Blue dotted lines show simulations with modified *k_e_* values. (A, B) *k_e_* values of 0.05 and 0.4 yield best fit simulations. (C, D) *k_e_* values of 0.5 (same as used in simulations 5, Table 2, Fig. 6C, D) lead to small improvement in simulations. *y* axis: normalized Dl levels; *x* axis: nuclei.
(TIF)

Figure S7 Analysis of model behavior using various randomly-generated parameter sets that fit the Dl gradient dynamics described in [15] shows the requirement of similar adjustments to reproduce the species-specific gradients. For 3 different parameter sets tested shown as examples (shown in Table S6), the species-specific gradients are reproduced with good fits after adjusting the values of *R*, *k_e_, k_Deg_* and *k_b_* in a similar way to the adjustments made for the representative parameter set that was selected in the main paper. A) wild type; (B) *D. busckii;* (C) *D. simulans;* (D) *D. sechellia*. Black solid lines indicate experimental gradients. Green dashed lines in (A) indicate simulations with unaltered parameter set values. Blue dashed lines indicate simulations after improving the parameter sets against *dl−/dl+* and *gyn* mutants (refer to Table 1) and using species-specific embryo morphology measurements. Red dashed lines indicate simulations using species-specific adjustments in relevant parameters (as shown in “bus 3”, “sim 5” and “sec 5” simulations from Table 2). Similar results were obtained with other 5 parameter sets tested.
(TIF)

Figure S8 Species comparison of the Dl and Cact proteins. Amino acid sequence comparison between *D. melanogaster* (mel), *D. simulans* (sim), *D. sechellia* (sec), *D. yakuba* (yak) and *D. santomea* (san) for relevant domains of Dl (A) and Cact (B). The genome sequence of *D. santomea* is not available, thus we partially sequenced the *D. santomea* Dl and Cact. Location for the following domains are shown: rel homology domain [32], [43]; nuclear localization signal (NLS) and nuclear export signals (NES1–4) [32], [33]; validated Cact serine phosphorylation sites and functional domain [36]; ankyrin repeats (red boxes) [44].
(TIF)

Figure S9 Model sensitivity analysis of parameters tested. (A–X) Each contour represents a drop of 0.01 in fit (square root of the sum of square differences between the gradient produced with the parameter values showed in the y and x axis and the gradient produced with wildtype *melanogaster* parameters), with exception for the following figures, in which each contour represents a drop of: (R) 0.011; (T) 0.0097; (V) 0.0062, (W) 0.012; (X) 0.0087. Dark Blue represents highest fit with *melanogaster* simulation, and lighter colors represent lower fits. Asterisk in (B) indicates error region due to large nuclear radius and small embryo size. The pairs of parameter values used for each mutant and species simulation is indicated with dots and letters: **a**, *D. melanogaster*; **b**, *D. busckii;* **c**, *D. simulans,* **d**, *D. sechellia;* **e**, *D. yakuba;* **f**, *D. santomea;* **g**, *dl−/dl+;* **h**, *ssm*; **i**, *gyn*.
(TIF)

Figure S10 Transformed graph of the Dl distribution from *D. santomea* (green line) and *D. yakuba* (blue line) where the *sna* activation threshold has been set to one, based on the activation patterns in hybrid embryos between the two species (for description of equivalent experiment, see [8]). Note that *D. santomea* has higher concentration levels of nuclear Dl when compared to *D. yakuba*.
(TIF)

Figure S11 Comparison between the original and the modified model. (A) Cross-section scheme representing normal flux between its two halves (arrows) and a single cell compartment named h. (B) Linearized half cross-section, with compartments 1 (ventral most cell) to n (dorsal most cell), and no-flux boundary conditions (crossed arrows). (C) A single compartment according to our modified model and Kanodia model (D). **w**: width of the cortical layer; **t**: thickness of the cross-section; **n**: number of compartments in a half (B,D) or full (C) DV cross-section; **L**: length of the embryo from the ventral to the dorsal midline.
(TIF)

Table S1 Term-by-term description of the model differential equations.
(DOCX)

Table S2 Description of the model's variables and parameters.
(DOCX)

Table S3 Parameters and equations used for the model nondimensionalization.
(DOCX)

Table S4 Genotype-specific parameter values used in Fig. 3 and Fig. S2.
(DOCX)

Table S5 Fit calculations for Figures 3–7.
(DOCX)

Table S6 Values of randomly selected parameter sets within the range of parameter cloud identified in [15] that were used to test the model behavior in response to changes in relevant parameters, as shown in Figure S7.
(DOCX)

Text S1 Kanodia model description.
(DOCX)

Text S2 Assumptions underlying *gyn* and *ssm* simulations with nondimensionalized equations.
(DOCX)

Text S3 Modified model description.
(DOCX)

Text S4 Cell-autonomous regime and compartmentalization.
(DOCX)

Text S5 Selection of parameters for detailed analysis of species-specific simulations.
(DOCX)
