# Supplementary figures and images for: Modeling of the Dorsal Gradient across Species Reveals Interaction between Embryo Morphology and Toll Signaling Pathway during Evolution
Source: PLoS Comput Biol. 2014 Aug 28;10(8):e1003807. doi: 10.1371/journal.pcbi.1003807 (PMC4148200; doi:10.1371/journal.pcbi.1003807)

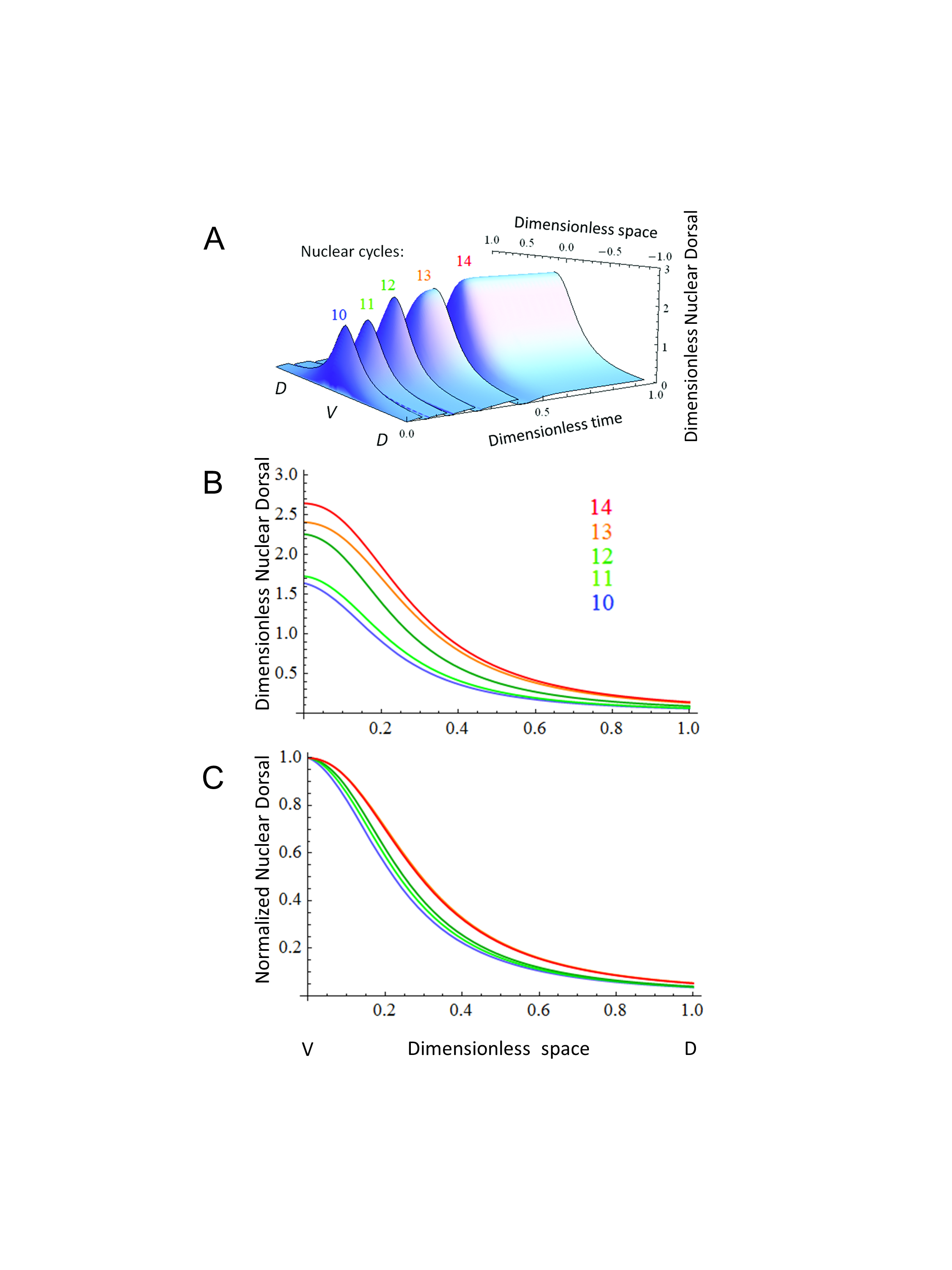

Supplement: Figure S1 — Reproduction of the original simulations from Kanodia et al. [15] in Mathematica. (A) 3D plots of wild type Dl gradient from nuclear cycle 10 to 14 (nc10–nc14). (B) Amplitude of the Dl gradient at the end of each interphase (see color code). (C) Dl gradient at the end of each interphase (same color code) normalized as a percentage of the highest nuclear Dl level, highlighting that the shape of the Dl gradient is conserved throughout development. Figures A, B and C should be compared to Figure 4A, 4B and 5D(ii), respectively, from Kanodia et al. original publication [15]. V: ventral midline; D: dorsal midline. (TIF) [file pcbi.1003807.s001.tif]

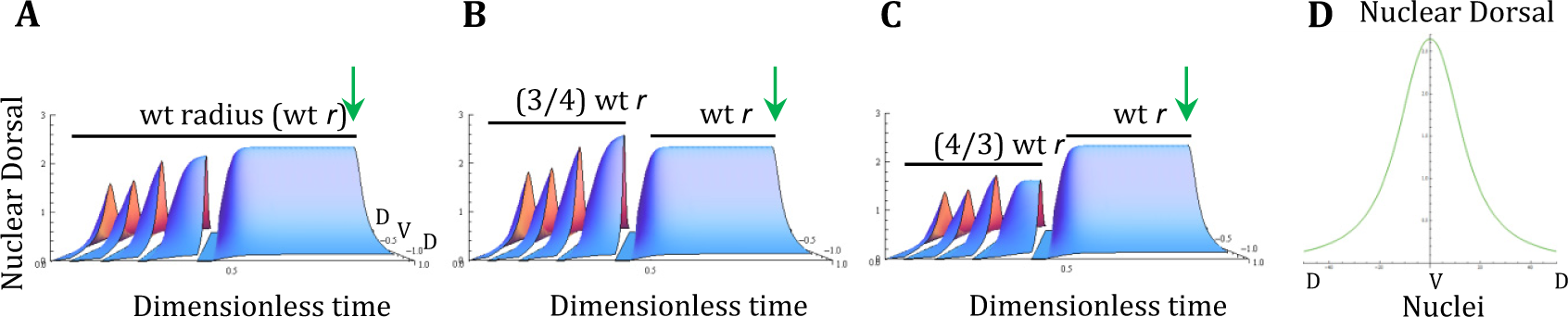

Supplement: Figure S2 — Simulations of changes in nuclear radius and the effect over Dl gradient shape. (A–C) Changes in nuclear radius (r) at nc10–13 affect the shape of the gradient at the respective cycles, but not at the last nuclear cycle. (D) Superposition of the gradients at the end of the last nuclear cycle from A–C (green arrows). V: ventral midline; D: dorsal midline. (TIF) [file pcbi.1003807.s002.tif]

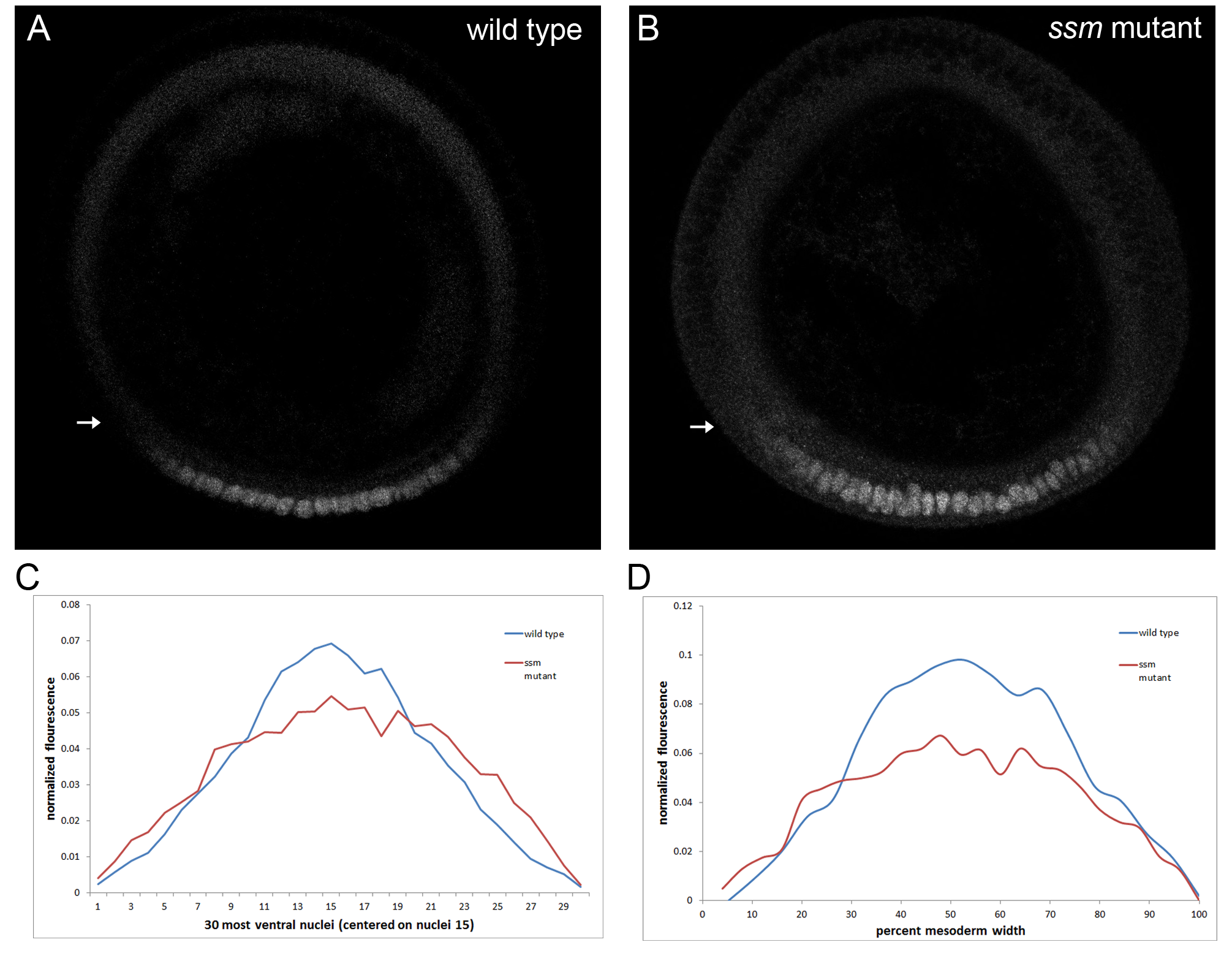

Supplement: Figure S3 — Plotting the Dl gradient along the entire DV axis or against percent mesoderm width reveal similar Dl distributions. (A) Wild type and (B) ssm blastoderm cross-sections stained for Dl protein (grey). Border of the 30 most ventral nuclei is indicated in one of the embryo sides (arrow, left side). Dorsal nuclei beyond this border have no detectable Dl signal with our method employed and the ratio of noise to signal is very high. (C) Normalization of the Dl gradient along the 30 most ventral nuclei for wild type (blue) and ssm (red), as used in this paper. (D) Dl gradient plotted against percent mesoderm width, where 19 nuclei comprise 100% mesoderm width in wild type D. melanogaster (blue) and 25 nuclei comprise 100% mesoderm width in ssm D. melanogaster (red). Note that differences in Dl distribution between wild type and ssm are clearly discernible (C, D). (TIF) [file pcbi.1003807.s003.tif]

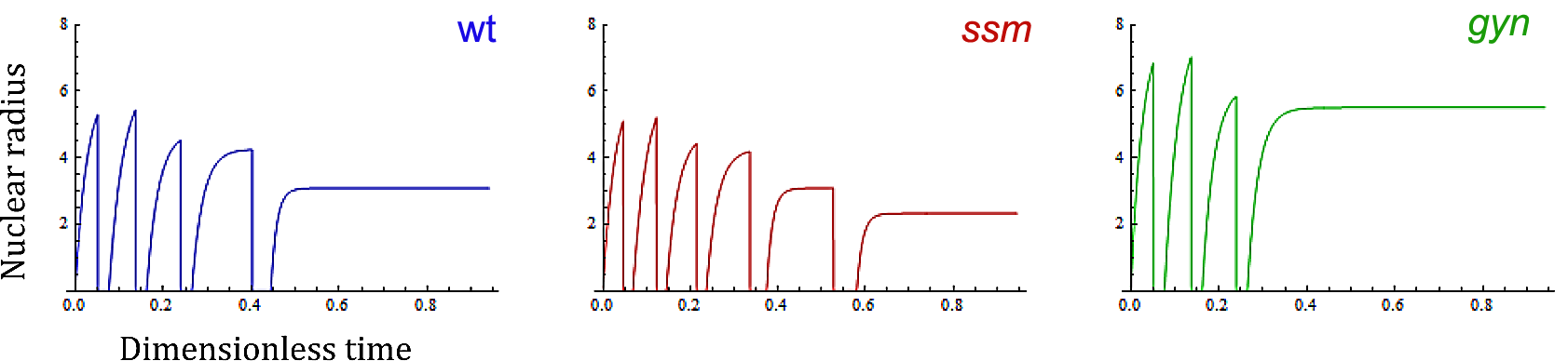

Supplement: Figure S4 — Time-dependent nuclear radius dynamics for wild type (blue), ssm (red) and gyn (green) used in Figure 3 and Figure S2. (TIF) [file pcbi.1003807.s004.tif]

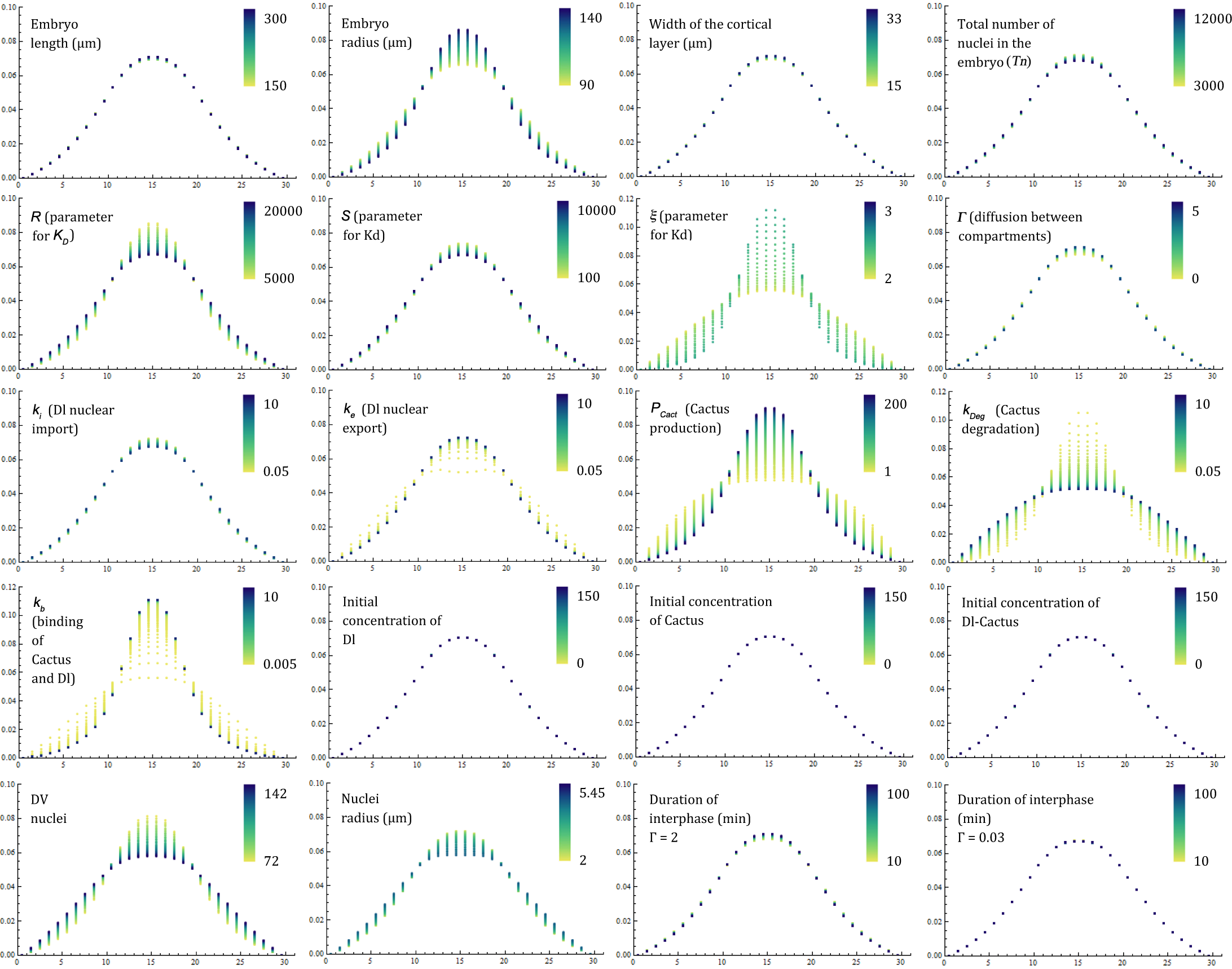

Supplement: Figure S5 — Individual influence of 19 parameters on the final shape of the Dl gradient. All graphs represent normalized nuclear Dl levels for the 30 most ventral cells of a cross-section at the end of nuclear cycle 14. With the exception of the parameter being manipulated, the values of the parameters used for all simulations are shown in Table 1, under D. melanogaster adjusted parameters. Note that the last two graphs show the effect of duration of interphase with distinct values of transport rates between compartments (Г). (TIF) [file pcbi.1003807.s005.tif]

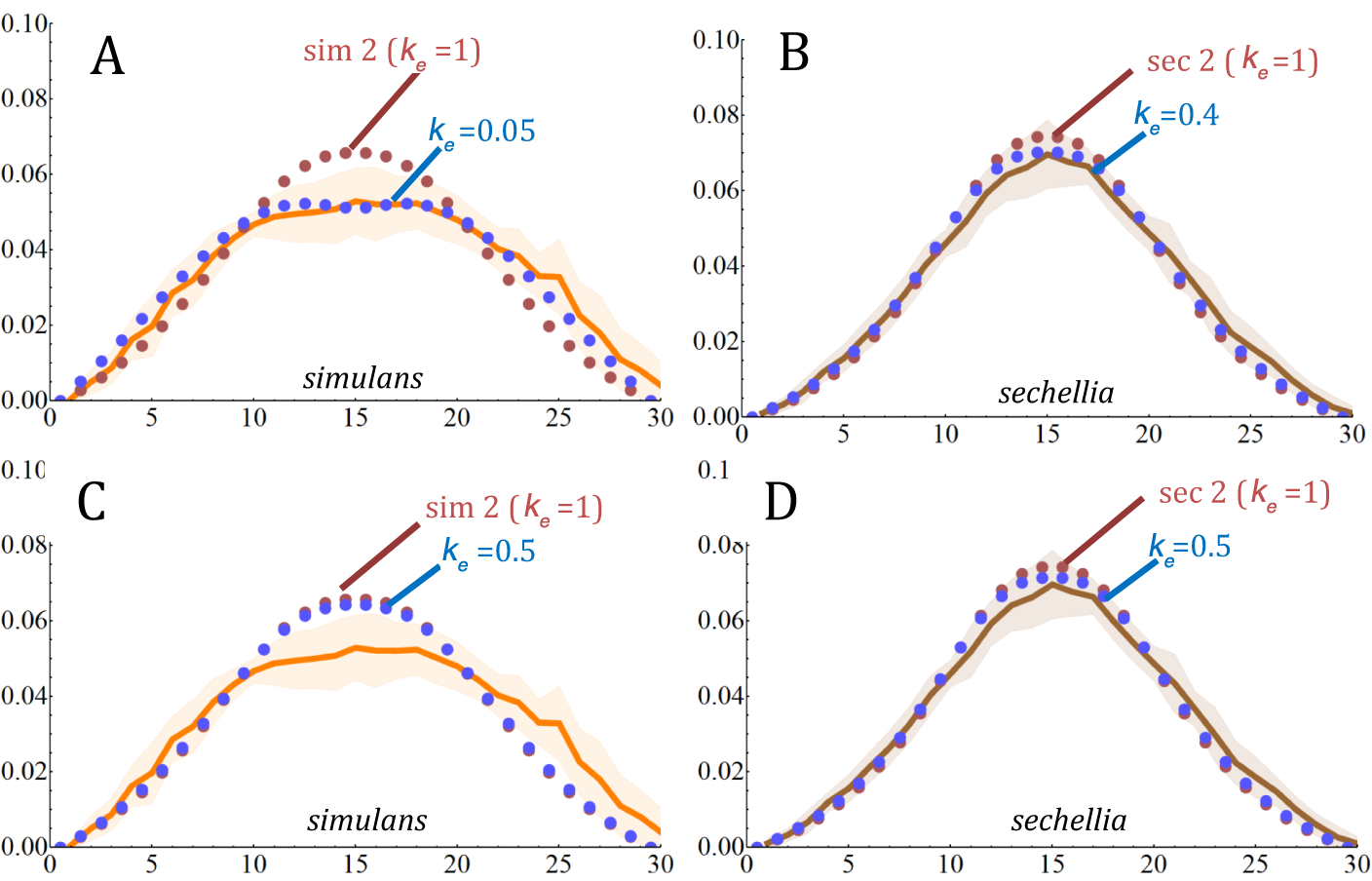

Supplement: Figure S6 — Simulations with changes in Dl nuclear export rates (ke) only in D. simulans and D. sechellia. (A–D) Experimental quantification of the Dl gradient (solid line; shadow represents average±SD) and model simulations (dotted lines) for D. simulans (A, C) and D. sechellia (B, D). Dark pink dotted lines represent simulations “sim 2” and “sec 2” (Table 2). Blue dotted lines show simulations with modified ke values. (A, B) ke values of 0.05 and 0.4 yield best fit simulations. (C, D) ke values of 0.5 (same as used in simulations 5, Table 2, Fig. 6C, D) lead to small improvement in simulations. y axis: normalized Dl levels; x axis: nuclei. (TIF) [file pcbi.1003807.s006.tif]

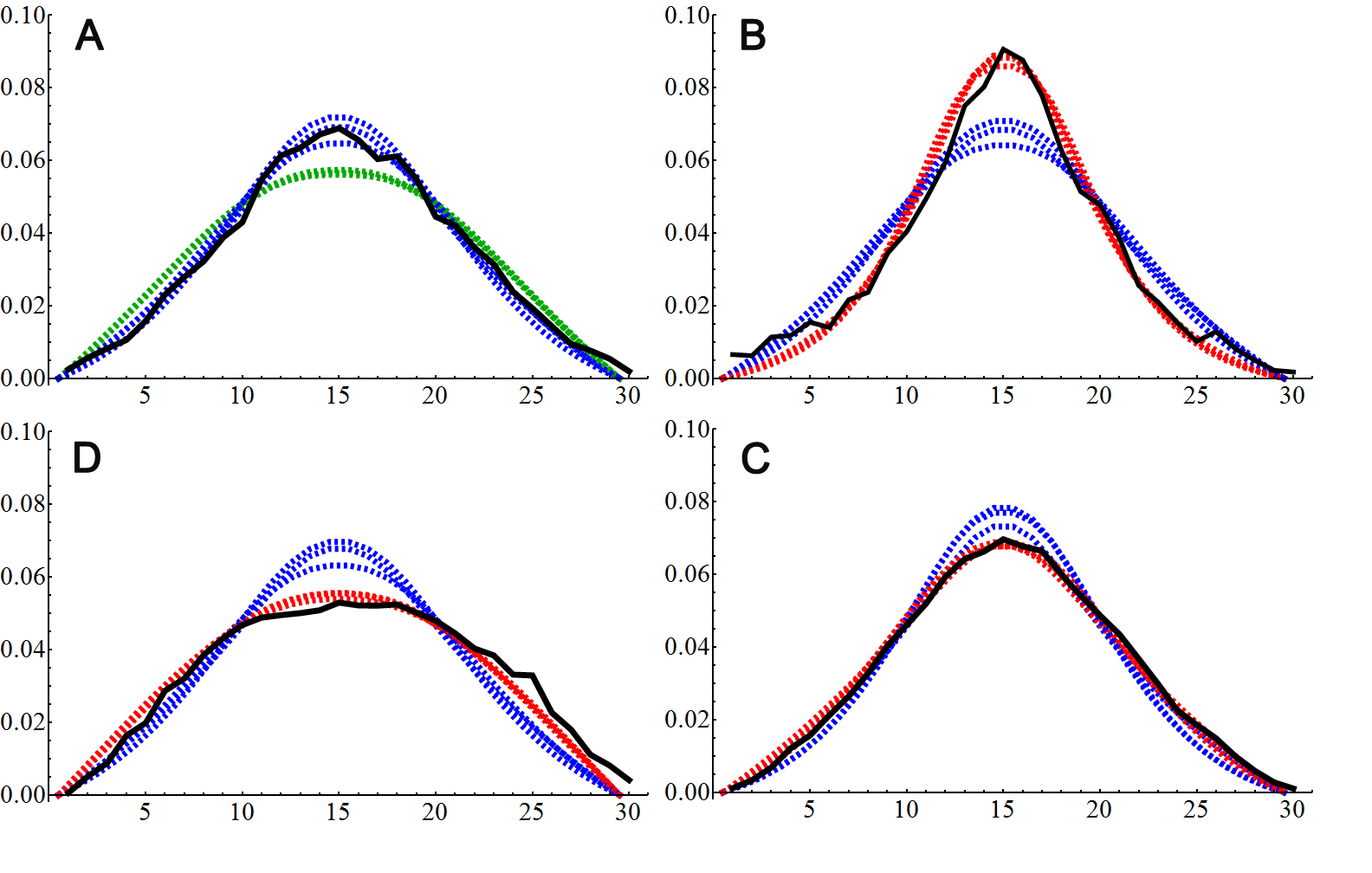

Supplement: Figure S7 — Analysis of model behavior using various randomly-generated parameter sets that fit the Dl gradient dynamics described in [15] shows the requirement of similar adjustments to reproduce the species-specific gradients. For 3 different parameter sets tested shown as examples (shown in Table S6), the species-specific gradients are reproduced with good fits after adjusting the values of R, ke, kDeg and kb in a similar way to the adjustments made for the representative parameter set that was selected in the main paper. A) wild type; (B) D. busckii; (C) D. simulans; (D) D. sechellia. Black solid lines indicate experimental gradients. Green dashed lines in (A) indicate simulations with unaltered parameter set values. Blue dashed lines indicate simulations after improving the parameter sets against dl−/dl+ and gyn mutants (refer to Table 1) and using species-specific embryo morphology measurements. Red dashed lines indicate simulations using species-specific adjustments in relevant parameters (as shown in “bus 3”, “sim 5” and “sec 5” simulations from Table 2). Similar results were obtained with other 5 parameter sets tested. (TIF) [file pcbi.1003807.s007.tif]

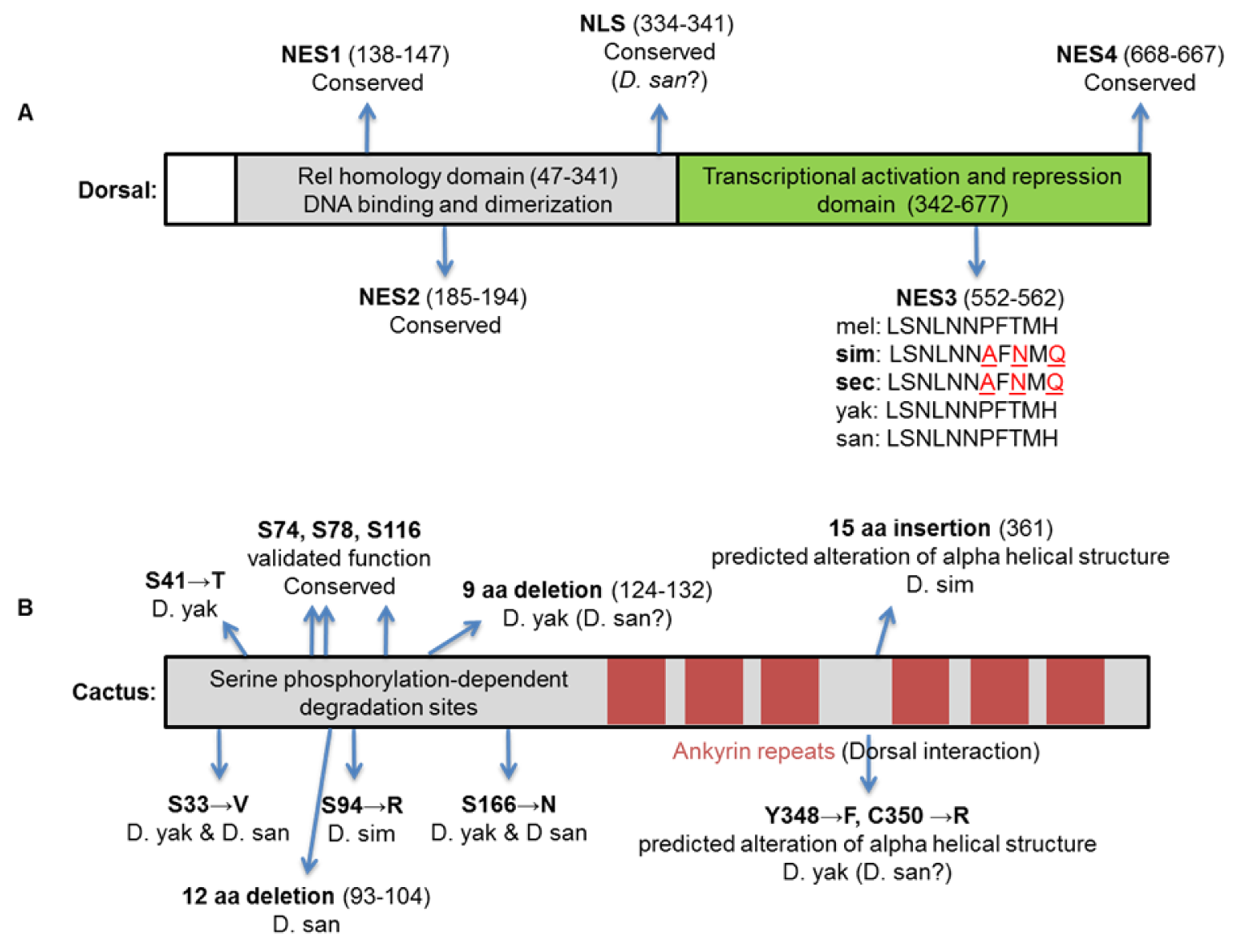

Supplement: Figure S8 — Species comparison of the Dl and Cact proteins. Amino acid sequence comparison between D. melanogaster (mel), D. simulans (sim), D. sechellia (sec), D. yakuba (yak) and D. santomea (san) for relevant domains of Dl (A) and Cact (B). The genome sequence of D. santomea is not available, thus we partially sequenced the D. santomea Dl and Cact. Location for the following domains are shown: rel homology domain [32], [43]; nuclear localization signal (NLS) and nuclear export signals (NES1–4) [32], [33]; validated Cact serine phosphorylation sites and functional domain [36]; ankyrin repeats (red boxes) [44]. (TIF) [file pcbi.1003807.s008.tif]

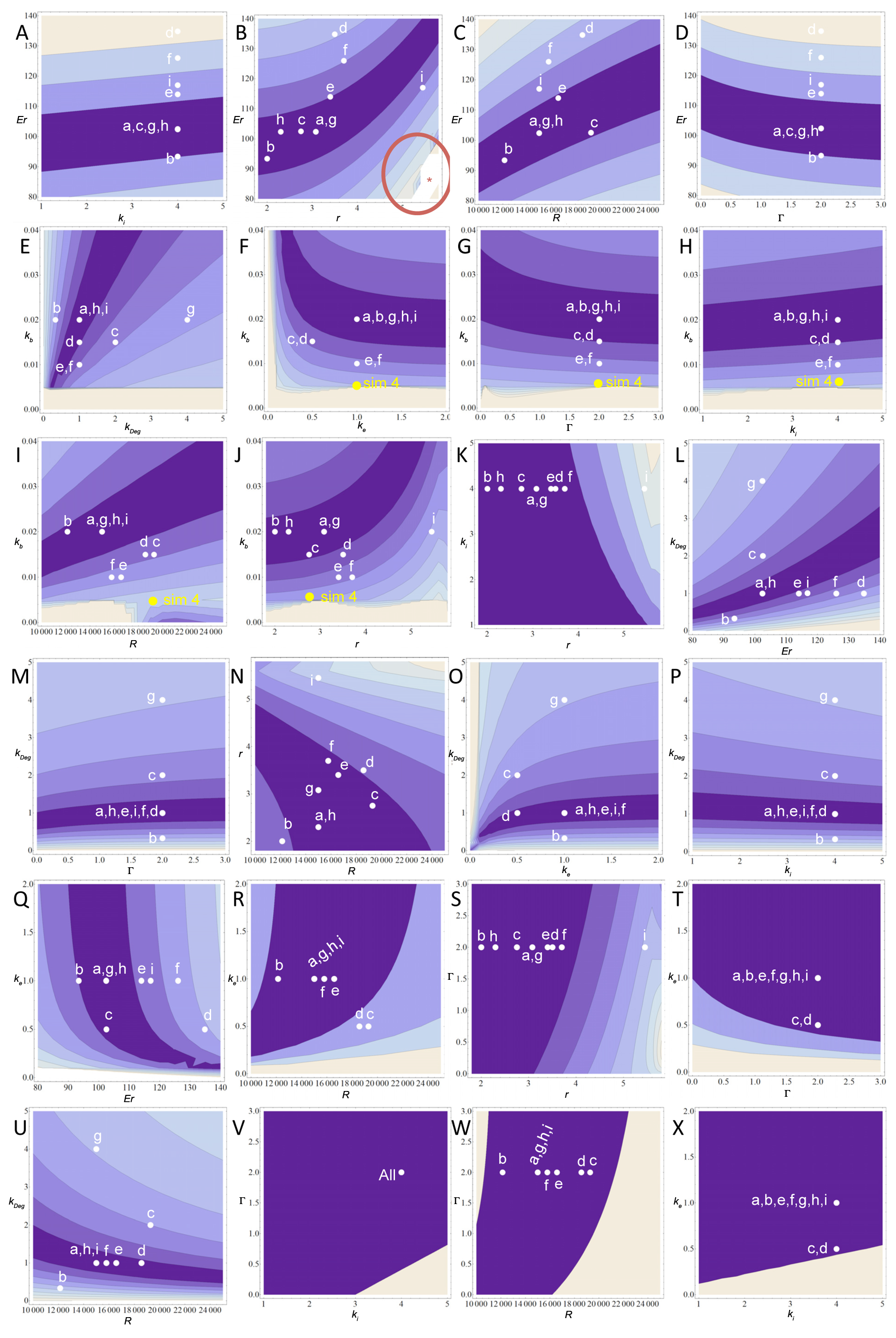

Supplement: Figure S9 — Model sensitivity analysis of parameters tested. (A–X) Each contour represents a drop of 0.01 in fit (square root of the sum of square differences between the gradient produced with the parameter values showed in the y and x axis and the gradient produced with wildtype melanogaster parameters), with exception for the following figures, in which each contour represents a drop of: (R) 0.011; (T) 0.0097; (V) 0.0062, (W) 0.012; (X) 0.0087. Dark Blue represents highest fit with melanogaster simulation, and lighter colors represent lower fits. Asterisk in (B) indicates error region due to large nuclear radius and small embryo size. The pairs of parameter values used for each mutant and species simulation is indicated with dots and letters: a, D. melanogaster; b, D. busckii; c, D. simulans, d, D. sechellia; e, D. yakuba; f, D. santomea; g, dl−/dl+; h, ssm; i, gyn. (TIF) [file pcbi.1003807.s009.tif]

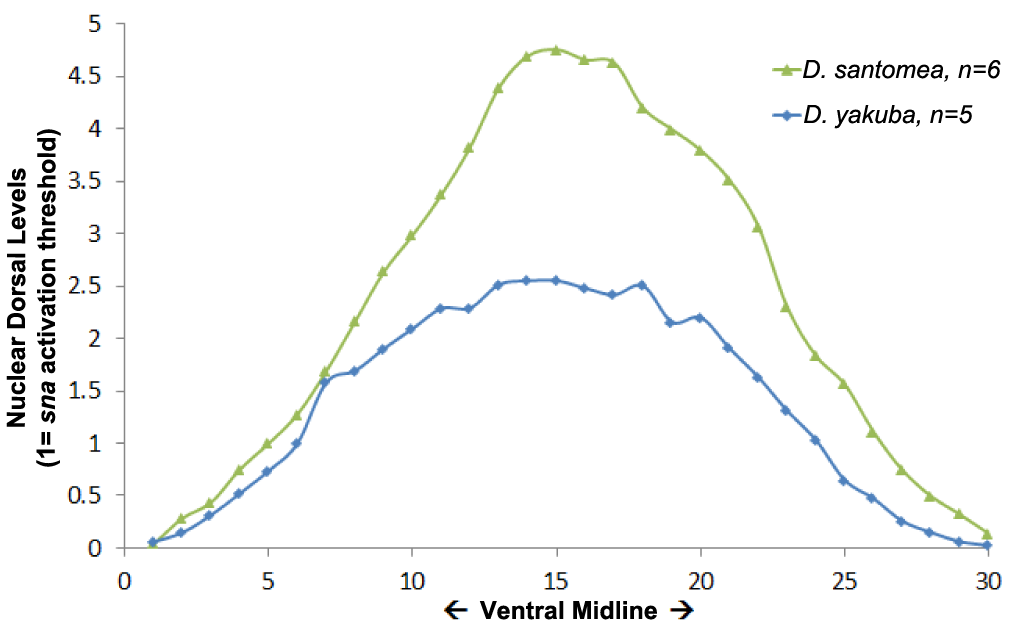

Supplement: Figure S10 — Transformed graph of the Dl distribution from D. santomea (green line) and D. yakuba (blue line) where the sna activation threshold has been set to one, based on the activation patterns in hybrid embryos between the two species (for description of equivalent experiment, see [8]). Note that D. santomea has higher concentration levels of nuclear Dl when compared to D. yakuba. (TIF) [file pcbi.1003807.s010.tif]

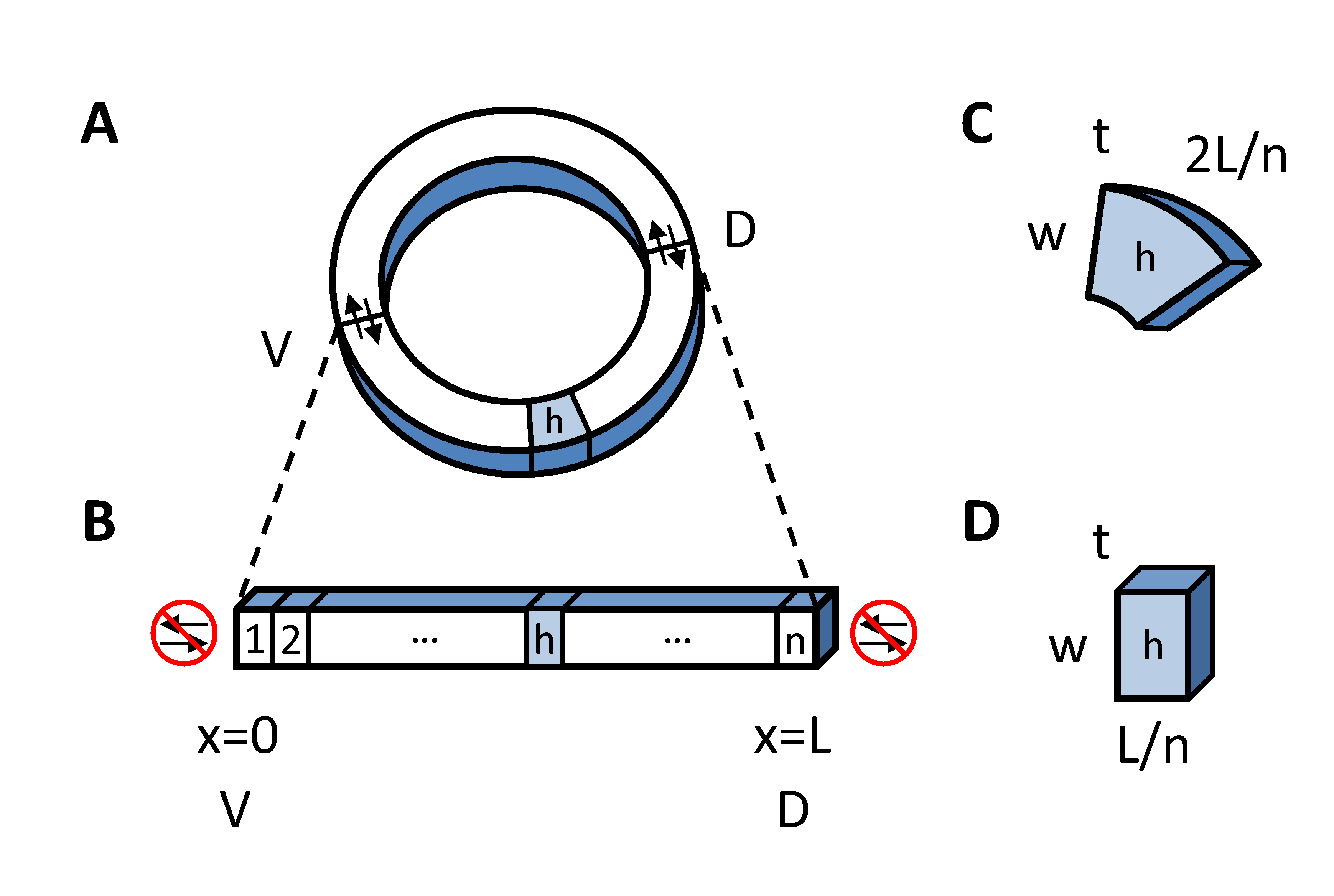

Supplement: Figure S11 — Comparison between the original and the modified model. (A) Cross-section scheme representing normal flux between its two halves (arrows) and a single cell compartment named h. (B) Linearized half cross-section, with compartments 1 (ventral most cell) to n (dorsal most cell), and no-flux boundary conditions (crossed arrows). (C) A single compartment according to our modified model and Kanodia model (D). w: width of the cortical layer; t: thickness of the cross-section; n: number of compartments in a half (B,D) or full (C) DV cross-section; L: length of the embryo from the ventral to the dorsal midline. (TIF) [file pcbi.1003807.s011.tif]
