# Supplementary material for: Modeling of the Dorsal Gradient across Species Reveals Interaction between Embryo Morphology and Toll Signaling Pathway during Evolution
Source: PLoS Comput Biol. 2014 Aug 28;10(8):e1003807. doi: 10.1371/journal.pcbi.1003807 (PMC4148200; doi:10.1371/journal.pcbi.1003807)
Supplement: Table S1 — Term-by-term description of the model differential equations. (DOCX) [file pcbi.1003807.s012.docx]

**Supporting Table S1.** Term-by-term description of the model differential equations.

| $\frac{d(V_{n}C_{Dl,n}^{h})}{dt}=$ | ${+A}_{n}k_{i}C_{Dl,c}^{h}$ | | ${-A}_{n}k_{e}C_{Dl,n}^{h}$ | |  |
| --- | --- | --- | --- | --- | --- |
| Time rate of change of the amount of Dl in the nucleus of the compartment h | Transport of Dl from the cytoplasm to the nucleus | | Transport of Dl from the nucleus to the cytoplasm | |  |
| $\frac{d(V_{c}C_{Dl,c}^{h})}{dt}=$ | ${+\Gamma A}_{m}(C_{Dl,c}^{h+1}-2C_{Dl,c}^{h}+C_{Dl,c}^{h-1})$ | $+k_{D}C_{Dl-cact,c}^{h}V_{c}$ | $-k_{b}C_{Dl,c}^{h}C_{cact,c}^{h}V_{c}$ | ${-A}_{n}k_{i}C_{Dl,c}^{h}$ | ${+A}_{n}k_{e}C_{Dl,n}^{h}$ |
| Time rate of change of the amount of Dl in the cytoplasm of the compartment h | Transport of Dl between the cytoplasm of the compartment h and the adjacent compartments | Dissociation of the Dl-Cact complex | Association of Dl and Cactus to form the Dl-Cact complex | Transport of Dl from the cytoplasm to the nucleus | Transport of Dl from the nucleus to the cytoplasm |
| $\frac{d(V_{c}C_{Dl-cact,c}^{h})}{dt}=$ | ${+\Gamma A}_{m}(C_{Dl-cact,c}^{h+1}-2C_{Dl-cact,c}^{h}+C_{Dl-cact,c}^{h-1})$ | | | $-k_{D}C_{Dl-cact,c}^{h}V_{c}$ | $+k_{b}C_{Dl,c}^{h}C_{cact,c}^{h}V_{c}$ |
| Time rate of change of the amount of Dl-Cact complex in the cytoplasm of the compartment h | Transport of Dl-Cact complex between the cytoplasm of the compartment h and the adjacent compartments | | | Dissociation of the Dl-Cact complex | Association of Dl and Cactus to form the Dl-Cact complex |
| $\frac{d(V_{c}C_{cact,c}^{h})}{dt}=$ | ${+\Gamma A}_{m}(C_{cact,c}^{h+1}-2C_{cact,c}^{h}+C_{cact,c}^{h-1})$ | $+k_{D}C_{Dl-cact,c}^{h}V_{c}$ | $-k_{b}C_{Dl,c}^{h}C_{cact,c}^{h}V_{c}$ | $+P_{cact}V_{c}$ | $-k_{Deg}C_{cact,c}^{h}V_{c}$ |
| Time rate of change of the amount of free Cactus in the cytoplasm of the compartment h | Transport of free Cactus between the cytoplasm of the compartment h and the adjacent compartments | Dissociation of the Dl-Cact complex | Association of Dl and Cactus to form the Dl-Cact complex | Production of Cactus | Degradation of Cactus |
| *k_D_ =* | *R / (S + x^ξ^)* | | |  |  |
| Space-dependent reaction rate constant for dissociation of the Dl-Cactus complex, representing the Toll signaling gradient | R and S determine the maximum value of *k_D_*, i.e., the amplitude of the Toll signaling gradient, while ξ represents the rate of decay of *k_D_* with an increase in x (the distance from the ventral midline along the DV axis). | | |  |  |
