# Supplementary material for: Modeling of the Dorsal Gradient across Species Reveals Interaction between Embryo Morphology and Toll Signaling Pathway during Evolution
Source: PLoS Comput Biol. 2014 Aug 28;10(8):e1003807. doi: 10.1371/journal.pcbi.1003807 (PMC4148200; doi:10.1371/journal.pcbi.1003807)
Supplement: Table S2 — Description of the model's variables and parameters. (DOCX) [file pcbi.1003807.s013.docx]

**Supporting Table S2.** Description of the model variables and parameters.

| Model Variables | |
| --- | --- |
| *x* | Distance from the ventral midline |
| $C_{Dl,n}^{h}$ | Concentration of Dl in the nucleus of compartment h |
| $C_{Dl,c}^{h}$ | Concentration of Dl in the cytoplasm of compartment h |
| $C_{Dl-cact,c}^{h}$ | Concentration of the Dl-Cactus complex in the cytoplasm of compartment h |
| $C_{cact,c}^{h}$ | Concentration of free Cactus in the cytoplasm of compartment h |
| Model Parameters | |
| *k_i_* | Rate at which Dl enters the nucleus |
| *k_e_* | Rate at which Dl exits the nucleus |
| $\Gamma$ | Transport rate of Dl, Dl-Cactus or Cactus between adjacent compartments |
| *k_b_* | Association rate of Dl and Cactus to form the Dl-Cactus complex |
| *P_Cact_* | Rate of Cactus production |
| *k_Deg_* | Rate of Cactus degradation |
| *k_D_* | Dissociation rate of the Dl-Cactus complex – the value of this parameter is space dependent as it represents the Toll signaling gradient |
| *R* | Parameters that describe the space-dependence of *k_D_* |
| *S* |  |
| $\xi$ |  |
|  |  |
| Time-dependent parameters | |
| *V_c_* | Volume of the cell compartment cytoplasm |
| *V_n_* | Volume of the cell compartment nucleus |
| *A_n_* | Surface area of the nucleus |
| *A_m_* | Surface area between two adjacent compartments |
