# Supplementary material for: Modeling of the Dorsal Gradient across Species Reveals Interaction between Embryo Morphology and Toll Signaling Pathway during Evolution
Source: PLoS Comput Biol. 2014 Aug 28;10(8):e1003807. doi: 10.1371/journal.pcbi.1003807 (PMC4148200; doi:10.1371/journal.pcbi.1003807)
Supplement: Table S3 — Parameters and equations used for the model nondimensionalization. (DOCX) [file pcbi.1003807.s014.docx]

**Supporting Table S3.** Parameters and equations used for the model nondimensionalization.

| Scaling factors | | | | | | |
| --- | --- | --- | --- | --- | --- | --- |
| *L* | Length of the embryo from the ventral to the dorsal midline | | | | | |
| *T* | Total developmental timing from the beginning of cycle 10 to the end of cycle 14 | | | | | |
| $C_{Dl-catc}^{o}$ | Concentration of Dl-Cactus complex at the beginning of cycle 10 | | | | | |
| $C_{catc}^{o}$ | Concentration of free Cactus at the beginning of cycle 10 | | | | | |
| $A_{n}^{14}$ | Area of the nucleus at the end cycle 14 | | | | | |
| $A_{m}^{14}$ | Surface area between two adjacent compartments at cycle 14 | | | | | |
| $V_{n}^{14}$ | Volume of nucleus at the end cycle 14 | | | | | |
| Dimensionless variables and time-dependent parameters | | | | | | |
| $\tau=\frac{t}{T}$ | | | $\bar{C}_{Dl,n}^{h}=\frac{C_{Dl,n}^{h}}{C_{Dl-cact}^{o}}$ | | $\bar{C}_{Dl-cact,c}^{h}=\frac{C_{Dl-cact,c}^{h}}{C_{Dl-cact}^{o}}$ | |
| $z=\frac{x}{L}$ | | | $\bar{C}_{Dl,c}^{h}=\frac{C_{Dl,c}^{h}}{C_{Dl-cact}^{o}}$ | | $\bar{C}_{cact,c}^{h}=\frac{C_{cact,c}^{h}}{C_{cact}^{o}}$ | |
| $\bar{V}_{n}=\frac{V_{n}}{V_{n}^{14}}$ | | $\bar{V}_{c}=\frac{V_{c}}{V_{n}^{14}}$ | | $\bar{A}_{n}=\frac{A_{n}}{A_{n}^{14}}$ | | $\bar{A}_{m}=\frac{A_{m}}{A_{m}^{14}}$ |
| Dimensionless parameters | | | | | | |
| $\sigma=\frac{A_{n}^{14}k_{i}}{V_{n}^{14}}T$ | Nuclear import | | | | | |
| $\mu=\frac{A_{n}^{14}k_{e}}{V_{n}^{14}}T$ | Nuclear export | | | | | |
| $\lambda=\frac{A_{m}^{14}\Gamma}{V_{n}^{14}}T$ | Transport across compartments | | | | | |
| $\gamma=k_{b}C_{cact}^{o}T$ | Association of Dl-Cactus complex | | | | | |
| $\alpha=k_{Deg}T$ | Degradation of Cactus | | | | | |
| $\psi=\frac{C_{Dl-cact}^{o}}{C_{cact}^{o}}$ | Relative levels of Dl and Cactus | | | | | |
| $\beta=\frac{RT}{L^{\xi}}$ | Characterization of the spatial pattern for Dl-Cactus dissociation rate | | | | | |
| $\varphi=\frac{S}{L^{\xi}}$ |  |  |  |  |  |  |
| $\xi$ |  |  |  |  |  |  |
