# Supplementary material for: Modeling of the Dorsal Gradient across Species Reveals Interaction between Embryo Morphology and Toll Signaling Pathway during Evolution
Source: PLoS Comput Biol. 2014 Aug 28;10(8):e1003807. doi: 10.1371/journal.pcbi.1003807 (PMC4148200; doi:10.1371/journal.pcbi.1003807)
Supplement: Table S4 — Genotype-specific parameter values used in Fig. 3 and Fig. S2. (DOCX) [file pcbi.1003807.s015.docx]

**Supporting Table S4**. Genotype-specific parameter values used in Fig. 3 and Fig. S2.

| Genotype | Last cycle before gastrulation | Number of DV nuclei at last cycle* | Nuclear radius at nc14 (µm) | Total duration of cycles (min) | | | | | |
| --- | --- | --- | --- | --- | --- | --- | --- | --- | --- |
|  |  |  |  | 10 | 11 | 12 | 13 | 14 | 15 |
| wt | 14 | 100 | 3.05 | 9 | 10 | 12 | 21 | 65 | - |
| *ssm* | 15 | 142 (100 * $\surd2$) | 2.3 | 9 | 10 | 12 | 18 | 27 | 55 |
| *gyn* | 13 | 72 (100 / $\surd2$) | 5.45 | 9 | 10 | 12 | 86 | - | - |

*In full embryo circumference. At each nuclear cycle, the number of DV nuclei is multiplied by $\surd2$ [15].
