# Supplementary material for: Modeling of the Dorsal Gradient across Species Reveals Interaction between Embryo Morphology and Toll Signaling Pathway during Evolution
Source: PLoS Comput Biol. 2014 Aug 28;10(8):e1003807. doi: 10.1371/journal.pcbi.1003807 (PMC4148200; doi:10.1371/journal.pcbi.1003807)
Supplement: Table S5 — Fit calculations for Figures 3–7. (DOCX) [file pcbi.1003807.s016.docx]

**Supporting Table S5.** Fit calculations for Figures 3-7.

| Simulation | Fit (x 10^-2^) with respective experimental data  (square root of square differences) |
| --- | --- |
| mel (Fig. 3D) | 3.19 |
| mel (Fig. 5B, pink) | 1.76 |
| mel (Fig. 5B, black) | 1.86 |
| *ssm* (Fig. 3E) | 1.68 |
| *ssm* 1 (Fig. 5D, pink) | 1.89 |
| *ssm* 2 (Fig. 5D, black) | 2.59 |
| gyn (Fig. 3F) | 8.65 |
| *gyn* (Fig. 5C, light pink dots) | 7.51 |
| *gyn* 1 (Fig. 5C, simulation 1) | 5.41 |
| *gyn* 2 (Fig. 5C, simulation 2) | 4.1 |
| *gyn* 3 (Fig. 5C, simulation 3) | 3.4 |
| *dl-/dl*+ (Fig. 4, light pink dots) | 4.31 |
| *dl-/dl*+ (Fig. 4, black; Fig. 5A pink) | 1.73 |
| *dl-/dl*+ (Fig. 4, dark pink dots) | 3.3 |
| *dl-/dl*+ (Fig. 5A, black) | 1.83 |
| bus (Fig. 6B, simulation 1) | 4.1 |
| bus (Fig. 6B, simulation 2) | 3.62 |
| bus (Fig. 6B, simulation 3) | 2.82 |
| sim (Fig. 6C, simulation 1) | 5.07 |
| sim (Fig. 6C, simulation 2) | 4.3 |
| sim (Fig. 6C, simulation 3) | 0.93 |
| sim (Fig. 6C, simulation 4) | 1.11 |
| sim (Fig. 6C, simulation 5) | 1.43 |
| sec (Fig. 6D, simulation 1) | 2.95 |
| sec (Fig. 6D, simulation 2) | 2.2 |
| sec (Fig. 6D, simulation 3) | 1.09 |
| sec (Fig. 6D, simulation 4) | 1.15 |
| sec (Fig. 6D, simulation 5) | 1.03 |
| yak (Fig. 7A, simulation 1) | 3.3 |
| yak (Fig. 7A, simulation 2) | 3.01 |
| yak (Fig. 7A, simulation 3) | 1.97 |
| yak (Fig. 7A, simulation 4) | 1.97 |
| san (Fig. 7B, simulation 1) | 3.26 |
| san (Fig. 7B, simulation 2) | 3.07 |
| san (Fig. 7B, simulation 3) | 1.18 |
| san (Fig. 7B, simulation 4) | 1.18 |
