# Supplementary material for: Modeling of the Dorsal Gradient across Species Reveals Interaction between Embryo Morphology and Toll Signaling Pathway during Evolution
Source: PLoS Comput Biol. 2014 Aug 28;10(8):e1003807. doi: 10.1371/journal.pcbi.1003807 (PMC4148200; doi:10.1371/journal.pcbi.1003807)
Supplement: Table S6 — Values of randomly selected parameter sets within the range of parameter cloud identified in [15] that were used to test the model behavior in response to changes in relevant parameters, as shown in Figure S7. (DOCX) [file pcbi.1003807.s017.docx]

**Supporting Table S6**. Values of randomly selected parameter sets within the range of parameter cloud identified in [15] that were used to test the model behavior in response to changes in relevant parameters, as shown in Supporting Figure S7.

|  | Set 1 | Set 2 | Set 3 | 1 Blue | 2 Blue | 3 Blue | 1 bus Red | 2 bus Red | 3 bus Red | 1 sim Red | 2 sim Red | 3 sim Red | 1 sec Red | 2 sec Red | 3 sec Red |
| --- | --- | --- | --- | --- | --- | --- | --- | --- | --- | --- | --- | --- | --- | --- | --- |
| *El* | * | * | * | * | * | * | 189 | 189 | 189 | 236 | 236 | 236 | 286.5 | 286.5 | 286.5 |
| *Er* | * | * | * | * | * | * | 93.4 | 93.4 | 93.4 | 102.5 | 102.5 | 102.5 | 134.85 | 134.85 | 134.85 |
| *Eh* | * | * | * | * | * | * | 23.4 | 23.4 | 23.4 | 30.1 | 30.1 | 30.1 | 29.6 | 29.6 | 29.6 |
| *Tn* | * | * | * | * | * | * | 6000 | 6000 | 6000 | 6000 | 6000 | 6000 | 7000 | 7000 | 7000 |
| *R* | 17637 | 199879 | 194442 | 17246 | 195443 | 190127 | **13969** | **158309** | **154003** | **22247** | **252122** | **245263** | **21385** | **242350** | **235757** |
| *S* | 3783 | 14384 | 1281 | 3735 | 14200 | 1265 | 3735 | 14200 | 1265 | 3735 | 14200 | 1265 | 3735 | 14200 | 1265 |
| *ξ* | 2.206 | 2.558 | 2.326 | 2.320 | 2.690 | 2.446 | 2.320 | 2.690 | 2.446 | 2.320 | 2.690 | 2.446 | 2.320 | 2.690 | 2.446 |
| *Г* | 0.518 | 1.129 | 4.852 | 34.674 | 75.536 | 324.711 | 34.674 | 75.536 | 324.711 | 34.674 | 75.536 | 324.711 | 34.674 | 75.536 | 324.711 |
| *k_i_* | 6.755 | 7.250 | 5.635 | 13.688 | 14.691 | 11.418 | 13.688 | 14.691 | 11.418 | 13.688 | 14.691 | 11.418 | 13.688 | 14.691 | 11.418 |
| *k_e_* | 0.361 | 4.732 | 2.663 | 0.826 | 10.830 | 6.095 | 0.826 | 10.830 | 6.095 | **0.413** | **5.415** | **3.048** | **0.413** | **5.415** | **3.048** |
| *P_Cact_* | 16.916 | 27.075 | 18.807 | 16.916 | 27.075 | 18.807 | 16.916 | 27.075 | 18.807 | 16.916 | 27.075 | 18.807 | 16.916 | 27.075 | 18.807 |
| *k_Deg_* | 2.976 | 5.927 | 2.062 | 2.976 | 5.927 | 2.062 | **0.331** | **0.395** | **0.458** | **11.906** | **23.708** | **8.247** | 2.976 | 5.927 | 2.062 |
| *k_b_* | 0.774 | 1.120 | 0.837 | 0.774 | 1.120 | 0.837 | 0.774 | 1.120 | 0.837 | **0.290** | **0.840** | **0.628** | **0.290** | **0.840** | **0.628** |
| *Dl0* | 36 | 36 | 36 | 36 | 36 | 36 | 36 | 36 | 36 | 36 | 36 | 36 | 36 | 36 | 36 |
| *Dl-Cact0* | 30 | 30 | 30 | 30 | 30 | 30 | 30 | 30 | 30 | 30 | 30 | 30 | 30 | 30 | 30 |
| *Cact0* | 36 | 36 | 36 | 36 | 36 | 36 | 36 | 36 | 36 | 36 | 36 | 36 | 36 | 36 | 36 |
| *r* | * | * | * | * | * | * | 2 | 2 | 2 | 2.75 | 2.75 | 2.75 | 3.5 | 3.5 | 3.5 |
| *n* | * | * | * | * | * | * | 86 | 86 | 86 | 98 | 98 | 98 | 102 | 102 | 102 |
| *t* | * | * | * | * | * | * | 65 | 65 | 65 | 65 | 65 | 65 | 65 | 65 | 65 |

Parameter sets 1-3 have a fit below 0.01 with the *D. melanogaster* wild type simulation shown in Figure 3D and reproduce the dynamics of the Dl gradient throughout nuclear cycles 10 to 14 as described by Kanodia *et al*. [15]. Blue (general adjustments) and red (species-specific adjustments) refer to simulations shown in Supporting Figure S7. Bold numbers indicate species-specific adjustments consistent with the ones used in Table 2. Abbreviations: **bus**, *D. busckii*; **sim**, *D. simulans*; **sec**, *D. sechellia*. For parameter abbreviations, see Table 1 and Supporting Figure S2. **El*, *Er*, *Eh*, *Tn*, *r*, *n*, and *t* are not free parameters and thus were not randomly generated from the original set. In simulations shown in blue (Supporting Figure S7), these parameters were adjusted to species-specific values.
