# Supplementary material for: Modeling of the Dorsal Gradient across Species Reveals Interaction between Embryo Morphology and Toll Signaling Pathway during Evolution
Source: PLoS Comput Biol. 2014 Aug 28;10(8):e1003807. doi: 10.1371/journal.pcbi.1003807 (PMC4148200; doi:10.1371/journal.pcbi.1003807)
Supplement: Text S1 — Kanodia model description. (DOCX) [file pcbi.1003807.s018.docx]

## Supporting Text S1

**Kanodia Model description.** For the first part of this paper, we used a minimally modified version of the Kanodia model, which is described in detail in [15].

**Zero-flux boundary conditions**. Kanodia simulates only half-circumference of the embryo mid cross-section and uses zero-flux boundary conditions at the most ventral and most dorsal cells (Supporting Figure S10B). In other words, there is no diffusion beyond these cells and they have only one neighboring cell compartment, with which their exchange rates are doubled. Hence, for all times:

At x=0 (ventral midline), $C_{sp,c}^{h=0}=C_{sp,c}^{h=2}$

At x=L (dorsal midline), $C_{sp,c}^{h=n+1}=C_{sp,c}^{h=n-1}$

sp $\in$ [Dl, Dl-Cactus, Cactus]

**Initial conditions.** For all x $\in$ [0, L]:

For nuclear cycle (nc) 10:

$C_{catc,c}^{h}= C_{catc,c}^{0}=$ *P_Cact_* / *k_Deg_*

$C_{Dl-catc,c}^{h}= C_{Dl-catc,c}^{o}$

$$C_{Dl,c}^{h}= C_{Dl,n}^{h}=0$$

For nc 11-14: based on concentration profiles established at the end of previous cycle (see [15] for details).

**Nondimensionalized equations**. The differential equations used by Kanodia are shown below, and a term-by-term explanations of each component of these equations is provided in Supporting Table S1. The kinetic relationships represented by the differential equations are accompanied by several unknown rate constants and properties related to the shape of the cellular compartments (summarized in Supporting Table S2). Some of these unknown values were combined into 9 dimensionless parameters (Supporting Table S3), while others were estimated with time-dependent equations. All equations and tables shown here are based on the supplementary information provided by Kanodia *et al*. [15].


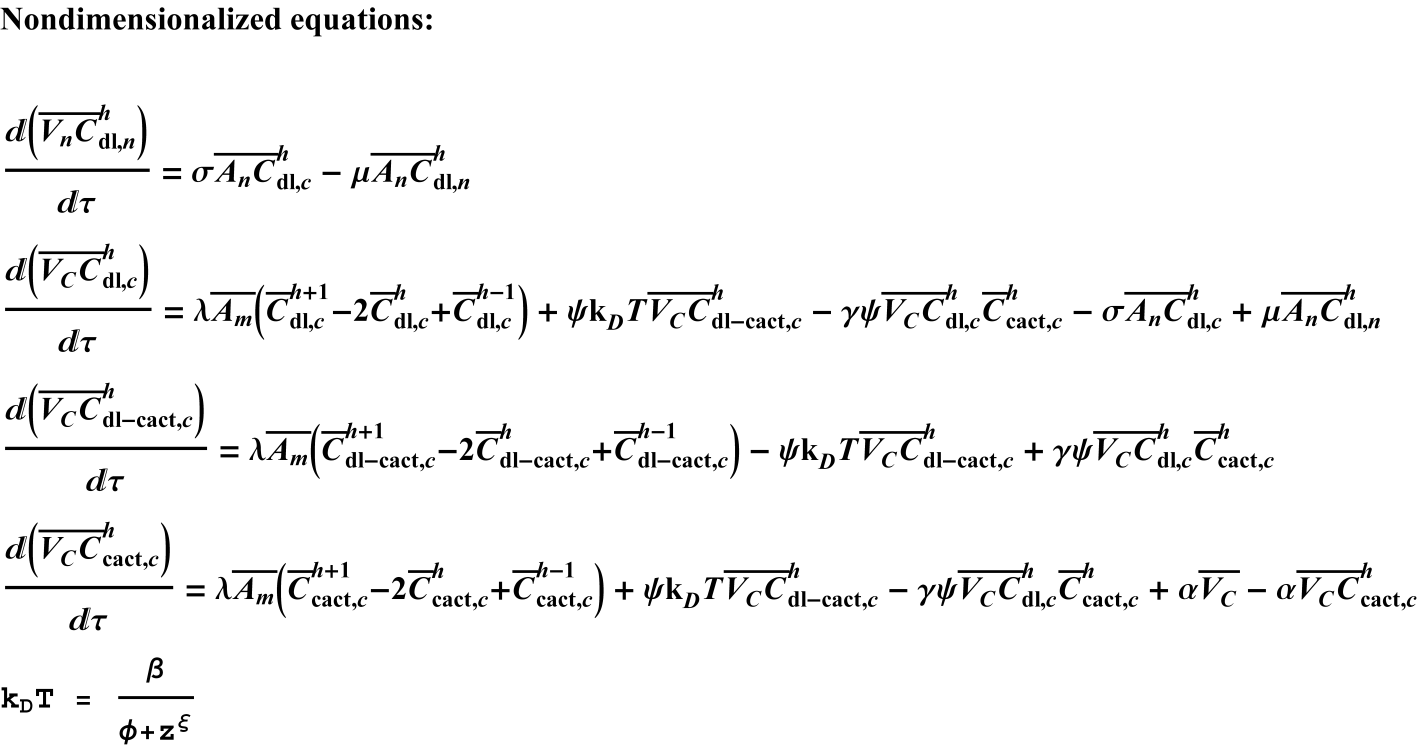


**Cell compartments.** To determine the volume of the cell compartments, Kanodia approximated the cortical region of the embryo as the space between two ellipsoids. He then divided the volume of the cortical region by 6000 cell compartments to estimate the volume of an individual compartment at nc14. The final value obtained by Kanodia was 865 µm^3^. This value doubles for each previous nuclear cycle, because the number of compartments halves. The nuclear volume, on the other hand, was determined by a time-dependent function based on the measurements by Gregor *et al*. [39] The volume of the cytoplasm was calculated by subtracting the nuclear volume from the compartment volume. Additionally, the surface area between two adjacent compartments is divided by $\surd2$ at the end of each nuclear cycle, because the width of the cortical layer is assumed to be constant by Kanodia *et al*.
