# Supplementary material for: Modeling of the Dorsal Gradient across Species Reveals Interaction between Embryo Morphology and Toll Signaling Pathway during Evolution
Source: PLoS Comput Biol. 2014 Aug 28;10(8):e1003807. doi: 10.1371/journal.pcbi.1003807 (PMC4148200; doi:10.1371/journal.pcbi.1003807)
Supplement: Text S2 — Assumptions underlying gyn and ssm simulations with nondimensionalized equations. (DOCX) [file pcbi.1003807.s019.docx]

## Supporting Text S2

**Assumptions underlying *gyn* and *ssm* simulations with nondimensionalized equations.** To simulate *ssm* and *gyn* using the nondimensionalized equations, we assumed that all genotypes had the same embryo size. The cell compartments of *ssm* at nc15 had half the volume of wildtype (wt) cell compartments at nc14, while *gyn* compartments at nc13 had the same volume as wt cell compartments at nc13. Also, developmental timing and nuclear radius were adjusted for *ssm* and *gyn* according to Supporting Table S4 and Supporting Figure S4. Developmental timing in haploid embryos was shown to be similar to wt until nc12, while in nc13/14, it becomes shorter and lasts about as long as the wt nc12/13 [40,41]. Limited information is available for triploid embryos. Their developmental timing was assumed to be normal until nc13 (when the nuclei stop dividing), and their nc13 was assumed to span the duration of wt nc13 and 14. Specific values used in the model are shown in Supporting Table S4. The nuclear radius prior to the last cycle is also unknown for the mutants. This measurement is challenging to be done even in wt embryos, because each cycle lasts about 10 minutes and nuclear size changes substantially from early-interphase to late-anaphase. However, the haploid nucleus seems to be similar to wt until nc14, suggesting that the extra division is responsible for nuclear reduction. The fact that the triploid nucleus is larger than wt at nc13 suggests that the triploid nucleus is generally larger than wt. Thus, the following assumptions were made: (1) the nuclear radius of *ssm* embryos is wt until its extra division, after which it adopts the value determined experimentally (2.3 µm); (2) *gyn* nuclear radius is given by an interpolation of wt values adjusted to the ratio *gyn*/wt nuclear radius at nc13 (5.45/4.2). A graphical representation of nuclear radius versus time for all genotypes is shown in Supporting Figure S4.
