# Supplementary material for: Modeling of the Dorsal Gradient across Species Reveals Interaction between Embryo Morphology and Toll Signaling Pathway during Evolution
Source: PLoS Comput Biol. 2014 Aug 28;10(8):e1003807. doi: 10.1371/journal.pcbi.1003807 (PMC4148200; doi:10.1371/journal.pcbi.1003807)
Supplement: Text S3 — Modified model description. (DOCX) [file pcbi.1003807.s020.docx]

## Supporting Text S3

**Modified model description.** In the original model, only a DV semi-circumference was modeled, which was approximated as a rectangular parallelepiped with no-flux boundary conditions (Supporting Fig. S10B). In combination with the assumption that the cortical layer width is constant throughout development, this simplification allowed Kanodia *et al.* to easily adjust the values of *A_m_* (Surface area between two adjacent compartments) and *V_c_* (Volume of the cell compartment cytoplasm) from nc 10 to 14. However, it is known that the cortical layer width is not constant throughout development [39], and that it is altered in *gyn* and *ssm* mutants, as well as in different species (Tables 1-3). As the width of the cortical layer increases, the error resulting from the linearization of the cross-section also increases. Thus, we developed a modified model in which a full DV cross-section was simulated, and the curved shape of the cell compartments was preserved (Supporting Fig. S10A, C).

Additionally, to maximize the simulation flexibility, the modified model simulates a single nuclear cycle, and all parameters shown in Table 1 can be manually adjusted. The volume of each cell compartment is calculated based on the size of the embryo, the width of the cortical layer and the total number of nuclei in the embryo, similarly to the method employed by Kanodia. Unlike the Kanodia model, the initial conditions are not adjusted according to previous cycles, because previous cycles are not simulated. Our decision to make this modification was based on our findings that early cycles do not affect the shape of the Dl gradient at nc14 (Supporting Figure S2), and it significantly reduces the computational requirements of the model. Also, we did not simulate time-dependent changes in nuclear radius. Instead, the nuclear radius is set to a constant value. All simulations shown in Figure 4 and Supporting Figure S5 and beyond were done using the modified model.
