# Supplementary material for: Modeling of the Dorsal Gradient across Species Reveals Interaction between Embryo Morphology and Toll Signaling Pathway during Evolution
Source: PLoS Comput Biol. 2014 Aug 28;10(8):e1003807. doi: 10.1371/journal.pcbi.1003807 (PMC4148200; doi:10.1371/journal.pcbi.1003807)
Supplement: Text S4 — Cell-autonomous regime and compartmentalization. (DOCX) [file pcbi.1003807.s021.docx]

## Supporting Text S4

**Cell-autonomous regime and compartmentalization.** Kanodia *et al*. [15] found that over 99% of their parameter sets satisfied two constraints of a steady state cell-autonomous system. These constraints are reproduced below and were obtained by calculating the steady state solution for the most ventral and most dorsal compartments assuming full compartmentalization, i.e. setting the value of the transport rate between compartments equal to zero ($\Gamma=0$).

Constraint 1: At ventral compartment: Nuclear Dl > Cytoplasmic Dl: 1 > $\frac{\mu}{\sigma}( 1+\frac{\gamma\varphi}{\beta} )$

Constraint 2: At dorsal compartment: Nuclear Dl < Cytoplasmic Dl: 1 < $\frac{\mu}{\sigma}( 1+\frac{\gamma\left( \varphi+1 \right)}{\beta} )$

In other words, if the values of µ, σ, γ, φ, and β fit those constraints, a Dl gradient is established even without diffusion between compartments, due to the space-dependent dissociation of Dl-Cact (*k_D_*). All of the parameter sets used here to simulate the gradients from *D. melanogaster* mutants and other *Drosophila* species fit those constraints, which means that our results do not exclude the possibility of compartmentalization in the system. However, we are interested in the shape of the Dl gradient established after the last syncytial division, instead of the formation of a generic Dl gradient. Thus, we tested two possible cell-autonomous scenarios with our model: (1) The Dl gradient formation is a cell-autonomous process from its onset, i.e. the final shape of the gradient can be reached without diffusion between adjacent compartments; or (2) Once the final Dl gradient is established, it reaches a cell-autonomous state and diffusion between compartments does not affect the shape of the gradient.

As shown in Figure 5 and Supporting Figure S5, the shape of the final gradient changes with different values of $\Gamma$. Supporting Figure S5 also shows that given high enough diffusion rates, the gradient gets steeper over time, which partly explains the larger impact of higher diffusion rates on *gyn* than on wt and *ssm* embryos, since the last nuclear cycle lasts longer for *gyn*. Thus, our model suggests that the establishment and maintenance of the final Dl gradient shape are not cell-autonomous. In summary, our results agree with Kanodia’s findings that diffusion between compartments is not required to produce a DV Dl gradient, but they suggest that the establishment and maintenance of the Dl gradient shape is not cell-autonomous, because diffusion is needed to reach the final shape of the gradient.

The unit conversion of *Г* discussed on page 23 was done as follows: To convert the transport rate constant *Г* (unit: length/time) to the more commonly used diffusion coefficient *D* (units: length^2^/time), we considered the transport term in our differential equations as a discretized Laplace operator in only one dimension, and obtained the following formula (see Supporting Table S2 and S3 for parameter abbreviations):

$$D=\frac{ГA_{m} L^{2}}{V_{c} n^{2}}$$
