# Supplementary material for: Modeling of the Dorsal Gradient across Species Reveals Interaction between Embryo Morphology and Toll Signaling Pathway during Evolution
Source: PLoS Comput Biol. 2014 Aug 28;10(8):e1003807. doi: 10.1371/journal.pcbi.1003807 (PMC4148200; doi:10.1371/journal.pcbi.1003807)
Supplement: Text S5 — Selection of parameters for detailed analysis of species-specific simulations. (DOCX) [file pcbi.1003807.s022.docx]

## Supporting Text S5

**Selection of parameters for detailed analysis of species-specific simulations.** After adjusting the parameter set for *D. melanogaster* experimental conditions, four main categories of parameters became apparent: (1) Non-free parameters (parameters that can be obtained by direct measurement, i.e. *El, Er, Eh*, *Tn*, *r, n, t*); (2) Parameters related to the arc-length of the mesoderm, which can be inferred by measurement (i.e. *R, S, ξ*); (3) Robust parameters that minimally affect the Dl gradient shape (i.e., *El, Eh*, *Dl0, Cact0, Dl-Cact0*; see Supporting Fig. S5); (4) Free parameters (i.e. *Г, k_i_, k_e_, P_Cact_, k_Deg_* and *k_b_*). For the species simulations, we selected free parameters with largest effects over the gradient shape (i.e. *k_e_, P_Cact_, k_Deg_* and *k_b_*). Due to the symmetrical effects of *P_Cact_* and *k_Deg_*, either of these two parameters could be tested interchangeably (results for *k_Deg_* are shown in the paper). Although parameters of the second category may produce some of the largest effects over the gradient shape and can reproduce the gradients of the species on their own (e.g. *R* and *ξ*), the values required for obtaining the fits with experimental data are in conflict with the measured arc-length of the mesoderm. In contrast, the modifications in the selected parameters are biologically meaningful and further supported by the sequence comparisons of the Cact and Dl from the species (See main text).
